# Macrophage Inactivation by Small Molecule Wedelolactone via Targeting sEH for the Treatment of LPS-Induced Acute Lung Injury

**DOI:** 10.1021/acscentsci.2c01424

**Published:** 2023-02-21

**Authors:** Juan Zhang, Min Zhang, Xiao-Kui Huo, Jing Ning, Zhen-Long Yu, Christophe Morisseau, Cheng-Peng Sun, Bruce D. Hammock, Xiao-Chi Ma

**Affiliations:** † College of Pharmacy, Dalian Medical University, Dalian 116044, China; ‡ Second Affiliated Hospital, Dalian Medical University, Dalian 116023, China; § School of Pharmaceutical Sciences, Health Science Center, Shenzhen University, Shenzhen 518061, China; ∥ Department of Entomology and Nematology, UC Davis Comprehensive Cancer Center, University of California, Davis, California 95616, United States

## Abstract

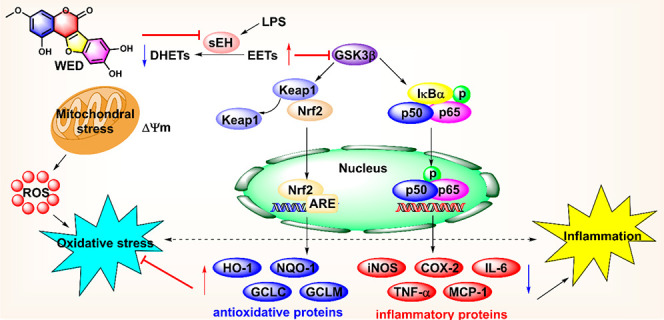

Soluble epoxide hydrolase (sEH) plays a critical role in inflammation by modulating levels of epoxyeicosatrienoic acids (EETs) and other epoxy fatty acids (EpFAs). Here, we investigate the possible role of sEH in lipopolysaccharide (LPS)-mediated macrophage activation and acute lung injury (ALI). In this study, we found that a small molecule, wedelolactone (WED), targeted sEH and led to macrophage inactivation. Through the molecular interaction with amino acids Phe362 and Gln384, WED suppressed sEH activity to enhance levels of EETs, thus attenuating inflammation and oxidative stress by regulating glycogen synthase kinase 3beta (GSK3β)-mediated nuclear factor-kappa B (NF-κB) and nuclear factor E2-related factor 2 (Nrf2) pathways *in vitro*. In an LPS-stimulated ALI animal model, pharmacological sEH inhibition by WED or sEH knockout (KO) alleviated pulmonary damage, such as the increase in the alveolar wall thickness and collapse. Additionally, WED or sEH genetic KO both suppressed macrophage activation and attenuated inflammation and oxidative stress *in vivo*. These findings provided the broader prospects for ALI treatment by targeting sEH to alleviate inflammation and oxidative stress and suggested WED as a natural lead candidate for the development of novel synthetic sEH inhibitors.

## Abstract 2

Targeting sEH with WED to enhance levels of EETs suppressed the macrophage activation via regulating GSK3β-mediated NF-κB and Nrf2 pathways, which resulted in the attenuation of LPS-induced ALI.

## Introduction

Macrophages are the first defense line of the innate immune system. They are distributed in many tissues of the human body, particularly in the respiratory tract.^1^ Macrophages function as scavengers to phagocytize bacteria, pathogens, and inhaled particulates and remove necrotic cells from the pulmonary environment to maintain homeostasis.^2,3^ As the phagocytosis of macrophages becomes dysfunctional, there is an increase in pathogens and apoptotic cells, which results in secondary necrosis and inflammation and aggravates the pathological course of diseases.^4^ Meanwhile, the presence of apoptotic cells in the lung maintains the proinflammatory phenotype of macrophages to decrease the ability for resolving the inflammation, which further drives the inflammatory profile in many pulmonary conditions.^3^

In the respiratory system, activated macrophages, whether caused by pathogens or exogenous substrates, e.g. lipopolysaccharide (LPS) and particulate matter, produce abundant proinflammatory factors, such as tumor necrosis factor-alpha (TNF-α) and interleukin-1 beta and 6 (IL-1β and IL-6), which contributes to the organ damage in inflamed tissues.^1,5,6^ Furthermore, the activation of macrophages induces the NADPH oxidase 2 (NOX2) and inducible nitric oxide synthase (iNOS) expression to promote the release of reactive oxygen and nitrogen species (ROS and RNS), thereby leading to mitochondrial damage and, finally, to the organ injury.^3^ Additionally, inflammation and oxidative stress mutually accelerate the damage.^7,8^ Therefore, blocking inflammation and oxidative stress serves as a vital strategy for the treatment of diseases of the respiratory system.^9,10^

Polyunsaturated fatty acids (PUFAs) belong to a number of ω-3 and ω-6 families and play an important role for maintaining the physiological health through their bioactive metabolites. The best studied of the polyunsaturated fatty acids is arachidonic acid (AA) and other PUFAs.^11−13^ AA exists in the form of phospholipids located in membranes and is released to the cytoplasm principally by the action of phospholipase A2.^14^ It is metabolized by three main pathways—cytochrome P450s (CYPs, e.g., CYP3A and CYP2J), cyclooxygenases (COXs, e.g., COX-2), and lipoxygenases (LOXs, e.g., 12-LOX), into bioactive derivatives.^14^ Among bioactive derivatives of AA, epoxy fatty acids (EpFAs) represented by epoxyeicosatrienoic acids (EETs), produced by several CYP450 oxidases (e.g., CYP2J and CYP2C), have received great attention from scientists because of their outstanding physiological effects, especially anti-inflammatory, antioxidant, and antalgesic activities.^14−16^ However, EETs are rapidly metabolized in the presence of epoxide hydrolases (EHs) represented by soluble epoxide hydrolase (sEH), which causes the loss of their multiple effects.^12,17−22^ sEH, encoded by *Ephx2*, is a bifunctional enzyme with 555 amino acid residues, and its C-terminal mediates the hydrolysis of bioactive epoxy fatty acids (EpFAs), such as EETs and epoxydocosapentaenoic acids (EDPs),^14,23^ while the role of the N-terminal phosphatase is poorly understood. Recently, sEH inhibition to enhance levels of EETs has become an attractive research strategy to treat diseases related to inflammation, such as lung injury and diabetes.^24−26^

An effective approach to discover innovative drugs involves probing natural products for possible drugs or leads because of their complex and changeable structures and multiple biological activities.^27,28^ Increasing evidence supports the therapeutic effects of natural products and traditional Chinese medicines, such as kuraninone, alisol B, (2*S*,3*S*)-britanicafanin A, 3β-hydroxy-25-anhydro-alisol F, and *Inula japonica*.^24,25,29−31^ Wedelolactone (WED), first isolated from *Wedelia calendulacea* by Govindachari and co-workers in 1956, is a polyphenol sharing a coumarin skeleton with a benzofuran moiety at C-3 and C-4.^32^ Emerging evidence demonstrates multiple pharmacological responses of WED, including anti-inflammatory, anticancer, and hepatoprotective effects, as well as the remission of Parkinson’s disease (PD) and kidney injury.^33−37^ Recent research focused on WED has indicated that it attenuates lung collagen deposition and fibrotic pathology in bleomycin-mediated pulmonary fibrosis.^38^ Similarly, WED protects bronchial epithelial cells from cigarette smoke extract-induced damage, as well.^39^ However, the protective mechanism and molecular target of WED in macrophage-mediated respiratory diseases, especially acute lung injury (ALI), still remains to be elucidated.

Herein, we investigated the ability of WED to reduce inflammation and oxidative stress in LPS-activated macrophages. To understand how it exerted the effect, we performed the target fishing experiments on the basis of the affinity chromatography to identify tentatively the cellular direct target of WED. We found that targeting sEH with WED enhanced levels of EETs to trigger the glycogen synthase kinase 3beta (GSK3β) inhibition, thereby leading to modulation of the nuclear factor-kappa B (NF-κB) and nuclear factor E2-related factor 2 (Nrf2) pathways. Meanwhile, pharmacological sEH inhibition by WED or sEH knockout (KO) exerted a significant therapeutic effect in the ALI animal model treated with LPS. Collectively, this study revealed that sEH served as a valuable target for the treatment of the inflammatory response and oxidative stress of ALI.

## Results

### WED Alleviated the Inflammatory Response *In Vitro* through the NF-κB Pathway

**Figure 1:**
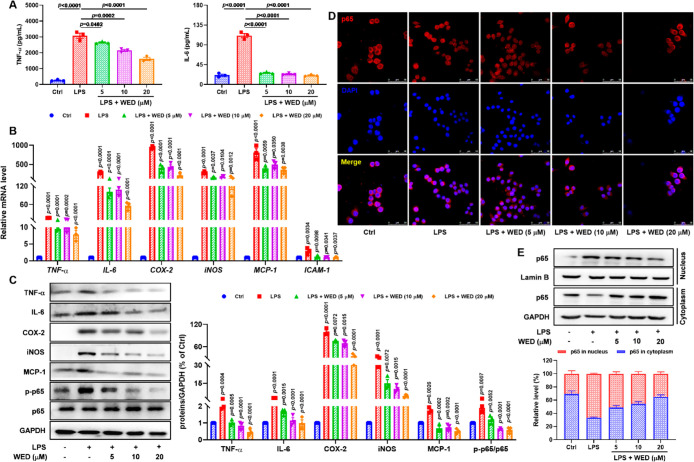
WED alleviated inflammatory responses *in vitro* through the NF-κB pathway. (A) WED suppressed the release of LPS-induced TNF-α (*p* < 0.0001; df = 4, 10; *F*-value = 143.3) and IL-6 (*p* < 0.0001; df = 4, 10; *F*-value = 238.1) in RAW264.7 cells (mean ± SEM, *n* = 3, one-way ANOVA). (B) WED downregulated mRNA levels of *TNF-α* (*p* < 0.0001; df = 4, 10; *F*-value = 71.86), *IL-6* (*p* < 0.0001; df = 4, 10; *F*-value = 147.1), *COX-2* (*p* < 0.0001; df = 4, 10; *F*-value = 79.7), *iNOS* (*p* < 0.0001; df = 4, 10; *F*-value = 33.1), *MCP-1* (*p* < 0.0001; df = 4, 10; *F*-value = 21.1), and *ICAM-1* (*p* = 0.0019; df = 4, 10; *F*-value = 9.6) in LPS-induced RAW264.7 cells (mean ± SEM, *n* = 3, one-way ANOVA). (C) WED inactivated the NF-κB pathway (*p* < 0.0001; df = 4, 10; *F*-value = 28.2) to downregulate expression levels of its target proteins TNF-α (*p* < 0.0001; df = 4, 10; *F*-value = 29.6), IL-6 (*p* < 0.0001; df = 4, 10; *F*-value = 31.1), COX-2 (*p* < 0.0001; df = 4, 10; *F*-value = 105.5), iNOS (*p* < 0.0001; df = 4, 10; *F*-value = 41.8), and MCP-1 (*p* < 0.0001; df = 4, 10; *F*-value = 23.7) in LPS-induced RAW264.7 cells (mean ± SEM, *n* = 3, one-way ANOVA). (D,E) WED suppressed the translocation of p65 to the nucleus analyzed by confocal microscopy (D) and Western blot (E) (mean ± SEM, *n* = 3, one-way ANOVA. For p65 in nucleus, *p* < 0.0001; df = 4, 10; *F*-value = 30.3. For p65 in cytoplasm, *p* = 0.0015; df = 4, 10; *F*-value = 10.2).

We investigated the inflammation resolving effect of WED by first determining the concentration of WED (5, 10, and 20 μM) in LPS-induced RAW264.7 macrophages as the cytotoxicity of 40 μM of WED (cell viability less than 80%, Figure S1). As described in Figure 1A, WED displayed a significantly anti-inflammatory effect in LPS-induced macrophages because it dose-dependently suppressed the release of inflammatory factors (e.g., TNF-α, IL-6, and NO; Figure S1). Furthermore, WED reversed the upregulation of LPS-induced inflammatory genes [e.g., *TNF-α*, *iNOS*, *IL-6*, *COX-2*, *monocyte chemoattractant protein-1* (*MCP-1*), and *intercellular cell adhesion molecule-1* (*ICAM-1*)], and proteins (e.g., TNF-α, COX-2, iNOS, IL-6, and MCP-1) through the NF-κB pathway in a dose-dependent manner (Figure 1B,C). The confocal microscopy results supported the observation that LPS exposure promoted the translocation of the transcript factor p65 to the nucleus, while WED administration suppressed this effect by WED administration (5, 10, and 20 μM; Figure 1D), which was further supported by Western blot to detect the level of p65 in the cytoplasmic and nuclear fractions (Figure 1E). These results supported the anti-inflammatory effect of WED *in vitro*.

### WED Alleviated the Oxidative Stress *In Vitro* through the Nrf2 Pathway

**Figure 2:**
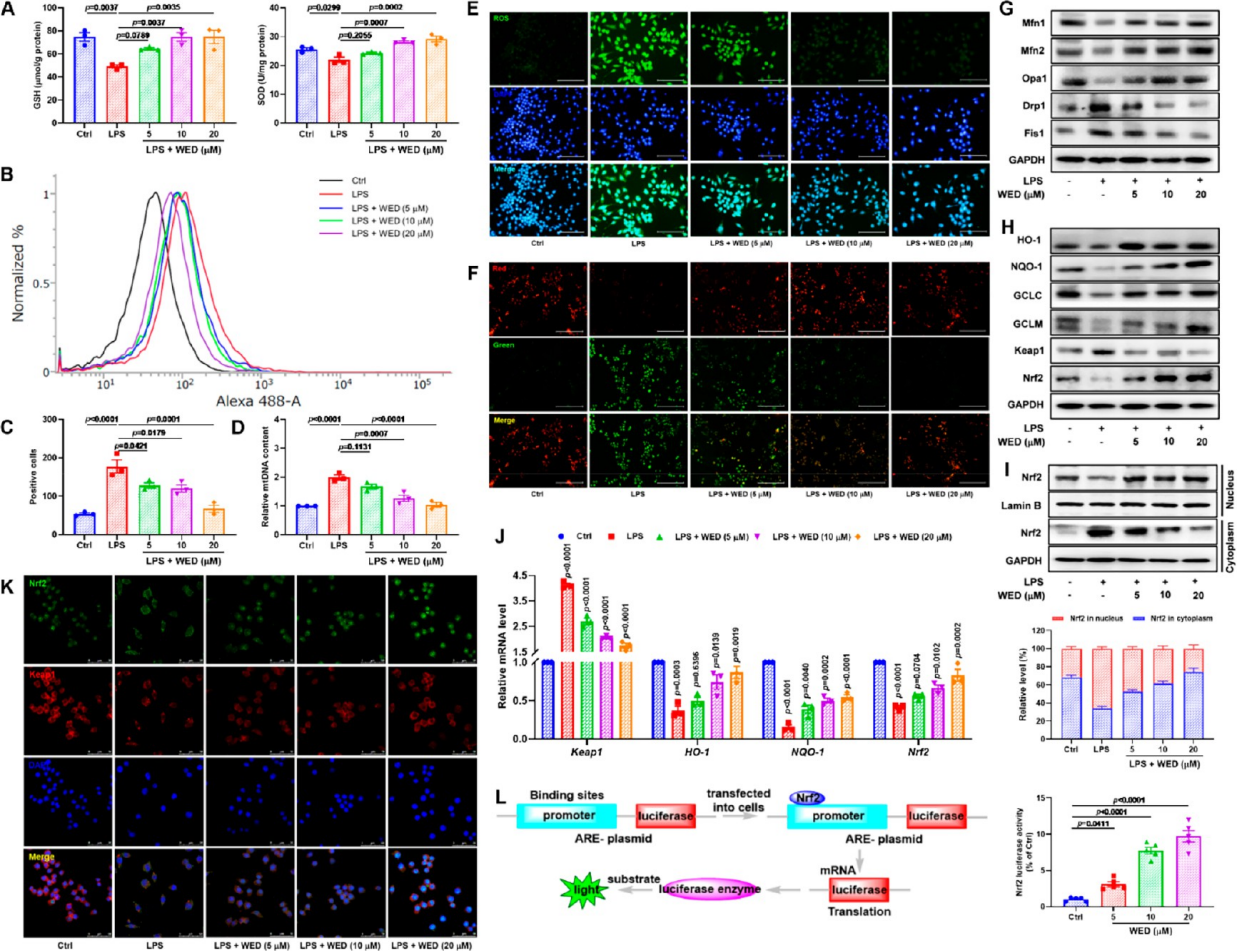
WED alleviated the oxidative reduction *in vitro* through the Nrf2 pathway. (A) WED enhanced levels of GSH (*p* = 0.0018; df = 4, 10; *F*-value = 9.8) and SOD (*p* = 0.0002; df = 4, 10; *F*-value = 17.1) in LPS-induced RAW264.7 cells (mean ± SEM, *n* = 3, one-way ANOVA). (B) The flow cytometry demonstrated that WED reduced the LPS-induced ROS-positive cells. (C) Quantitative data of ROS-positive cells in LPS-stimulated RAW264.7 cells treated with WED (mean ± SEM; *n* = 3; one-way ANOVA; *p* < 0.0001; df = 4, 10; *F*-value = 24.0). (D) WED reversed the increase of mtDNA content in LPS-induced RAW264.7 cells (mean ± SEM; *n* = 3; one-way ANOVA; *p* < 0.0001; df = 4, 10; *F*-value = 27.6). (E) The fluorometric analysis indicated that WED suppressed the ROS production. (F) WED reversed the effect of LPS-mediated mitochondrial membrane potential. (G) WED regulated expressions of proteins Mfn1, Mfn2, Op1, Drp1, and Fis1 involved in the mitochondrial fusion and fission. (H) WED activated the Nrf2 pathway to regulate expression levels of HO-1, NQO-1, GCLC, GCLM, Nrf2, and Keap1. (I,K) WED suppressed the translocation of Nrf2 to the nucleus analyzed by Western blot (I) (mean ± SEM, *n* = 3, one-way ANOVA. For Nrf2 in nucleus, *p* < 0.0001; df = 4, 10; *F*-value = 38.9. For Nrf2 in cytoplasm, *p* = 0.0001; df = 4, 10; *F*-value = 18.67) and the confocal microscopy (K). (J) WED regulated mRNA levels of genes *Keap1* (*p* < 0.0001; df = 4, 10; *F*-value = 202.5), *HO-1* (*p* = 0.0002; df = 4, 10; *F*-value = 16.5), *NQO-1* (*p* < 0.0001; df = 4, 10; *F*-value = 84.5), and *Nrf2* (*p* < 0.0001; df = 4, 10; *F*-value = 30.0) involved in the Nrf2 pathway (mean ± SEM, *n* = 3, one-way ANOVA). (L) The luciferase assay demonstrated the activation of WED against the Nrf2 receptor (mean ± SEM; *n* = 5; one-way ANOVA; *p* < 0.0001; df = 3, 16; *F*-value = 65.6).

Inflammatory response and oxidative stress always coexist in diseases, and they mutually promote and accelerate the progression of diseases. This knowledge stimulated us to examine the antioxidant effect of WED in LPS-induced macrophages. Described in Figure 2A, LPS treatment reduced the glutathione (GSH) level and superoxide dismutase (SOD) activity when compared with the control group, while WED (5, 10, and 20 μM) administration reversed these changes. The result of flow cytometry described in Figure 2B,C also suggested that WED suppressed the increase of LPS-induced ROS-positive cells, which was supported by a fluorescence experiment stained by 2′,7′-dichlorofluorescein diacetate (DCFH-DA) (Figure 2E). The production of ROS usually originates from mitochondrial damage. Thus, we subsequently detected the mitochondrial DNA (mtDNA) content, mitochondrial membrane potential, and genes and proteins responsible for the fusion and fission in the mitochondria. The mtDNA and JC-1 staining revealed that LPS resulted in the increase of the mtDNA copy number and mitochondrial membrane potential (Figure 2D,F), whereas these changes were reversed after administration of WED (5, 10, and 20 μM) in combination with effects of WED on the regulation of mitofusin 1 and 2 (Mfn1 and 2) and optic atrophy 1 (Opa1), which are responsible for the mitochondrial fusion, dynamin-related protein 1 (Drp1), and fission 1 (Fis1), involved in the mitochondrial fission (Figures 2G and S2), thereby requiring the protective effect of WED toward LPS-mediated mitochondrial damage. Nrf2 is the key transcript factor involved in the mitochondrial redox system; thus, we also assayed for the regulatory effect of WED toward this pathway. Pretreatment of WED (5, 10, and 20 μM) upregulated expressions of heme oxygenase-1 (HO-1), glutamate–cysteine ligase catalytic subunit (GCLC), glutamate–cysteine ligase modifier subunit (GCLM), NAD(P)H/quinone oxidoreductase 1 (NQO-1), and Nrf2; downregulated the kelch-like ECH-associated protein l (Keap1) expression; and regulated mRNA levels of their corresponding genes, such as *HO-1*, *Keap1*, *NQO-1*, and *Nrf2*, in LPS-induced macrophages (Figures 2H,J and S3). Additionally, WED treatment promoted the traffic of Nrf2 to the nucleus in LPS-induced macrophages (Figure 2I,K). This observation was supported by the results of a luciferase experiment showing the agonistic effect of WED toward the Nrf2 receptor (Figure 2L). These findings are consistent with the antioxidant effects of WED in macrophages treated with LPS.

### WED Attenuated the Pulmonary Damage in LPS-Induced ALI Mice

**Figure 3:**
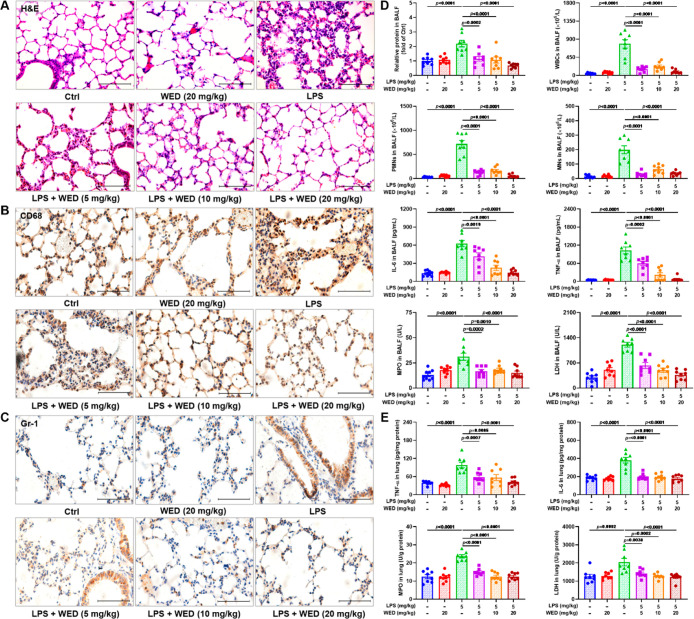
WED attenuated the course of ALI in LPS-induced ALI mice. (A) Representative H&E staining plots. (B) Representative CD68 staining plots. (C) Representative Gr-1 staining plots. (D) WED attenuated the infiltration of proteins (*p* < 0.0001; df = 5, 42; *F*-value = 11.3), WBCs (*p* < 0.0001; df = 5, 42; *F*-value = 36.2), PMNs (*p* < 0.0001; df = 5, 42; *F*-value = 57.8), and MNs (*p* < 0.0001; df = 5, 42; *F*-value = 32.1) to the pulmonary alveoli and reduced the production of IL-6 (*p* < 0.0001; df = 5, 42; *F*-value = 31.8) and TNF-α (*p* < 0.0001; df = 5, 42; *F*-value = 41.2) and the activity of MPO (*p* < 0.0001; df = 5, 42; *F*-value = 9.8) and LDH (*p* < 0.0001; df = 5, 42; *F*-value = 27.7) in LPS-induced ALI mice (mean ± SEM, *n* = 8, one-way ANOVA). (E) WED reduced the production of IL-6 (*p* < 0.0001; df = 5, 42; *F*-value = 23.8) and TNF-α (*p* < 0.0001; df = 5, 42; *F*-value = 14.2) and the activity of MPO (*p* < 0.0001; df = 5, 42; *F*-value = 27.6) and LDH (*p* < 0.0001; df = 5, 42; *F*-value = 7.9) in the lung of LPS-induced ALI mice (mean ± SEM, *n* = 8, one-way ANOVA).

The intratracheal instillation of LPS was used to construct the ALI mouse model for investigating the protective effect of WED *in vivo*. As described in Figure 3A–C, LPS treatment (5 mg/kg) increased the alveolar wall thickness and collapse. It also resulted in the activation of macrophages and the infiltration of neutrophils because of an increase in clusters of differentiation 68 (CD68)- and granulocyte-differentiation antigen-1 (Gr-1)-positive cells compared with the control group, while these changes were reversed in LPS-induced ALI mice after WED (5, 10, and 20 mg/kg) treatment. Furthermore, we found that WED treatment significantly reduced the number of white blood cells (WBCs), polymorphonuclear leukocytes (PMNs), and mononuclear leukocytes (MNs) in the bronchoalveolar lavage fluid (BALF) (Figure 3D), as well as levels of TNF-α and IL-6 and the activities of myeloperoxidase (MPO) and lactate dehydrogenase (LDH) in the BALF and lungs of LPS-induced ALI mice (Figure 3D,E). Collectively, these data reveal that WED attenuates the pathological lung injury.

### WED Attenuated LPS-Stimulated Inflammation and Oxidative Stress *In Vivo*

**Figure 4:**
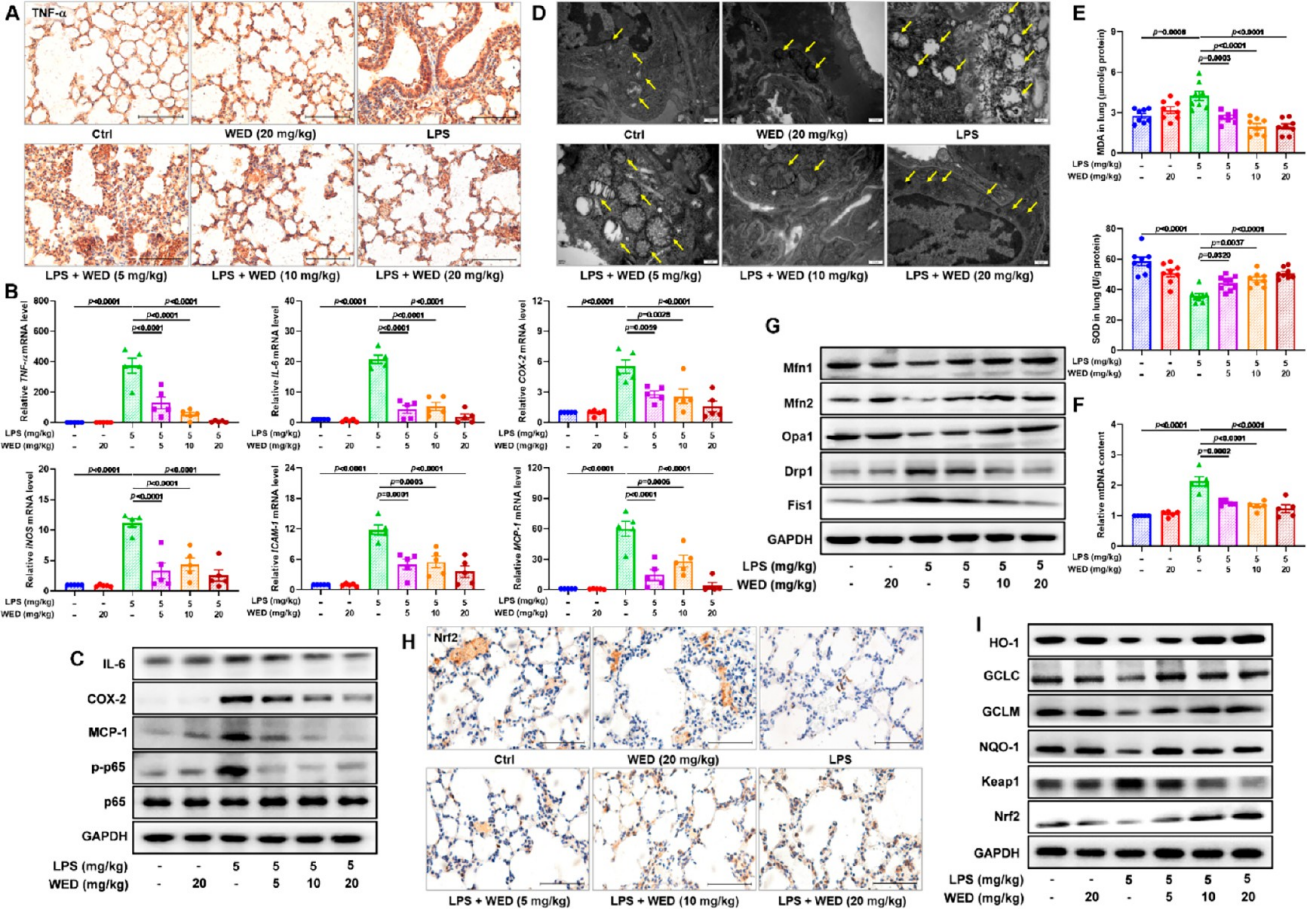
WED attenuated the inflammation and oxidative stress *in vivo*. (A) Representative TNF-α staining plots. (B) Effects of WED against inflammatory genes *TNF-α* (*p* < 0.0001; df = 5, 24; *F*-value = 30.5), *IL-6* (*p* < 0.0001; df = 5, 24; *F*-value = 56.7), *COX-2* (*p* < 0.0001; df = 5, 24; *F*-value = 12.8), *iNOS* (*p* < 0.0001; df = 5, 24; *F*-value = 21.0), *ICAM-1* (*p* < 0.0001; df = 5, 24; *F*-value = 21.4), and *MCP-1* (*p* < 0.0001; df = 5, 24; *F*-value = 25.5) in LPS-induced ALI mice (mean ± SEM, *n* = 5, one-way ANOVA). (C) WED suppressed the NF-κB pathway to downregulate expression levels of its target proteins IL-6, COX-2, and MCP-1. (D) Representative scanning electron microscope plots. (E) Effects of WED toward the MDA level (*p* < 0.0001; df = 5, 42; *F*-value = 13.4) and the SOD activity (*p* < 0.0001; df = 5, 42; *F*-value = 15.4) in LPS-induced ALI mice (mean ± SEM, *n* = 8, one-way ANOVA). (F) WED reversed the increase of the mtDNA content in LPS-induced ALI mice (mean ± SEM; *n* = 8; one-way ANOVA; *p* < 0.0001; df = 5, 24; *F*-value = 20.3). (G) WED regulated expressions of proteins Mfn1, Mfn2, Op1, Drp1, and Fis1 involved in the mitochondrial fusion and fission in LPS-induced ALI mice. (H) Representative Nrf2 staining plots. (I) WED activated the Nrf2 pathway to regulate expression levels of HO-1, NQO-1, GCLC, GCLM, Nrf2, and Keap1 in LPS-induced ALI mice.

The TNF-α staining results suggest that WED pretreatment (5, 10, and 20 mg/kg) suppressed the increase of TNF-α positive cells in lungs of LPS-induced ALI mice (Figure 4A), as well as the *TNF-α* mRNA level (Figure 4B). Moreover, WED treatment downregulated mRNA and protein levels of IL-6, iNOS, ICAM-1, COX-2, and MCP-1 through the inactivation of the NF-κB pathway because of inhibition of the phosphorylation of p65 (Figures 4B,C and S4A).

The ROS imbalance leads to oxidative stress, which contributes to the mtDNA damage in the ALI and leads to the abnormal mitochondrial ultrastructure. Figures 4E,F and S4B revealed that the LPS challenge led to a striking increase in the malondialdehyde (MDA) level and the mtDNA content and a remarkable decrease in the levels of GSH and SOD, which were significantly suppressed after WED treatment (5, 10, and 20 mg/kg). The mitochondrial architecture in the control group (Figure 4D) was characterized by isolated, small, and rounded mitochondria with a clear ridge, while the LPS challenge led to mitochondrial swelling and the disappearance of a clear ridge (Figure 4D). The appearance of ridges returned to normal with WED treatment (5, 10, and 20 mg/kg). Meanwhile, we found that WED treatment regulated the expression of proteins and genes involved in mitochondrial fusion (e.g., Mfn1, Op1, and Mfn2) and fission (e.g., Drp1 and Fis1) in LPS-induced ALI, thus allowing the recovery of the mitochondrial function (Figures 4G and S5). Additionally, WED treatment reversed LPS-mediated oxidative stress *in vivo* by activating the Nrf2 pathway, which was supported by the experiments on the basis of the Nrf2 staining, Western blot, and PCR (Figures 4H,I and S6). These aforementioned results revealed anti-inflammatory and antioxidant effects of WED *in vivo*.

### sEH Served as the Direct Cellular Target of WED in the Anti-Inflammation and Antioxidation

**Figure 5:**
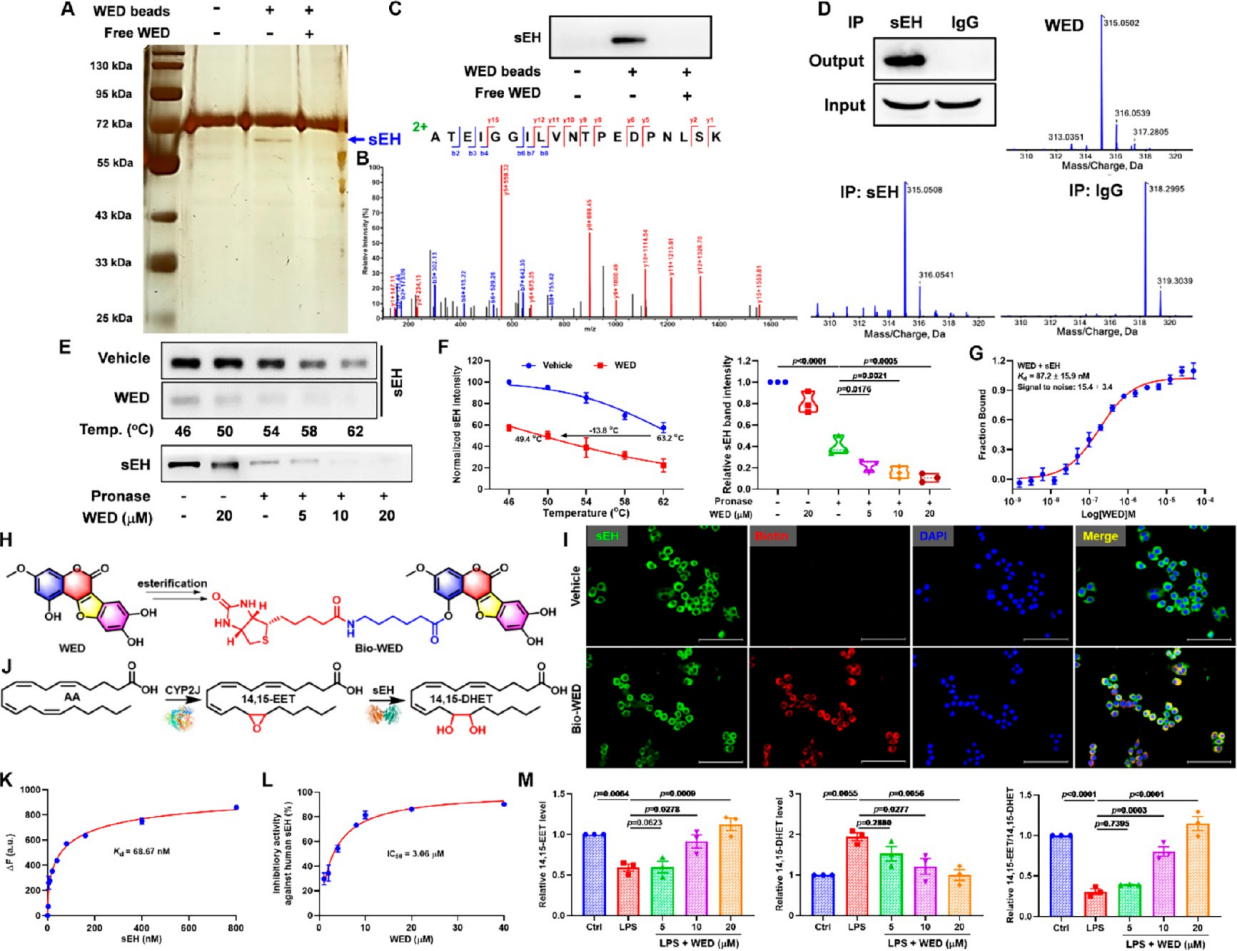
sEH served as the direct target of WED in anti-inflammation and antioxidation. (A) Identification of the cellular target of WED using the pull-down technology on the basis of WED-coupled Sepharose 6B beads and LC–MS/MS analysis. (B) The LC-MS/MS plot of sEH. (C) The binding protein was detected by Western blot. (D) The IP-MS analysis indicated the interaction of WED with sEH. (E) CETSA and DARTS results. (F) Quantitative data of CETSA and DARTS (mean ± SEM; *n* = 3; one-way ANOVA; *p* < 0.0001; df = 5, 12; *F*-value = 100.2). (G) The MST result of WED with sEH (mean ± SEM, *n* = 3). (H) The scheme of Bio-WED. (I) The colocation of WED with sEH detected by the fluorescence microscope. (J) The CYP/sEH-mediated AA metabolism pathway. (K) The binding capability of WED with sEH detected by the fluorescence-based binding assay (mean ± SEM, *n* = 3). (L) The inhibitory effect of WED against the human sEH activity detected by the system of human recombinant sEH-mediated hydrolysis of the substrate PHOME (mean ± SEM, *n* = 3). (M) WED suppressed the sEH activity (14,15-EET/14,15-DHET, *p* < 0.0001; df = 4, 10; *F*-value = 55.89) analyzed by levels of 14,15-EET (*p* = 0.0003; df = 4, 10; *F*-value = 15.1) and its corresponding diol (*p* = 0.0034; df = 4, 10; *F*-value = 8.2) in LPS-mediated RAW264.7 cells (mean ± SEM, *n* = 3, one-way ANOVA).

To discover the direct cellular target of WED, we used epoxy-activated Sepharose beads coupled with WED to perform the target fishing experiment. A distinct protein band appeared at ∼63 kDa after the sliver staining (Figure 5A) and was identified as sEH on the basis of the LC-MS/MS analysis (Figure 5B), which was further verified by Western blot using its corresponding antibody (Figure 5C). Meanwhile, the immunofluorescent colocalization analysis demonstrated the direct binding of WED and sEH (Figure 5H,I), which was supported by immunoprecipitation (IP)-MS, cellular thermal shift assay (CETSA), and drug affinity responsive target stability (DARTS) results (Figure 5D–F). The MST and fluorescence-based binding assay results revealed the outstanding binding affinity of WED and sEH with *K*_d_ values of 87.2 and 68.7 nM (Figure 5G,K), respectively. sEH as the functional protein with a hydrolase activity in the C-terminal plays a critical role in the AA metabolism (Figure 5J); we found that WED significantly suppressed the human and mouse sEH activity in the enzyme level (Figures 5L and S9). In addition, WED enhanced the level of the sEH substrate 14,15-EET and reduced its diol level in LPS-induced macrophages (Figure 5M). The ratio of 14,15-EET and 14,15-DHET also reflected the inhibitory effect of WED in the cell level (Figure 5M). These results suggest the direct binding effect of WED with sEH.

### sEH Knockdown and Rescue Abolished Anti-Inflammatory and Antioxidant Effects of WED *In Vitro*

**Figure 6:**
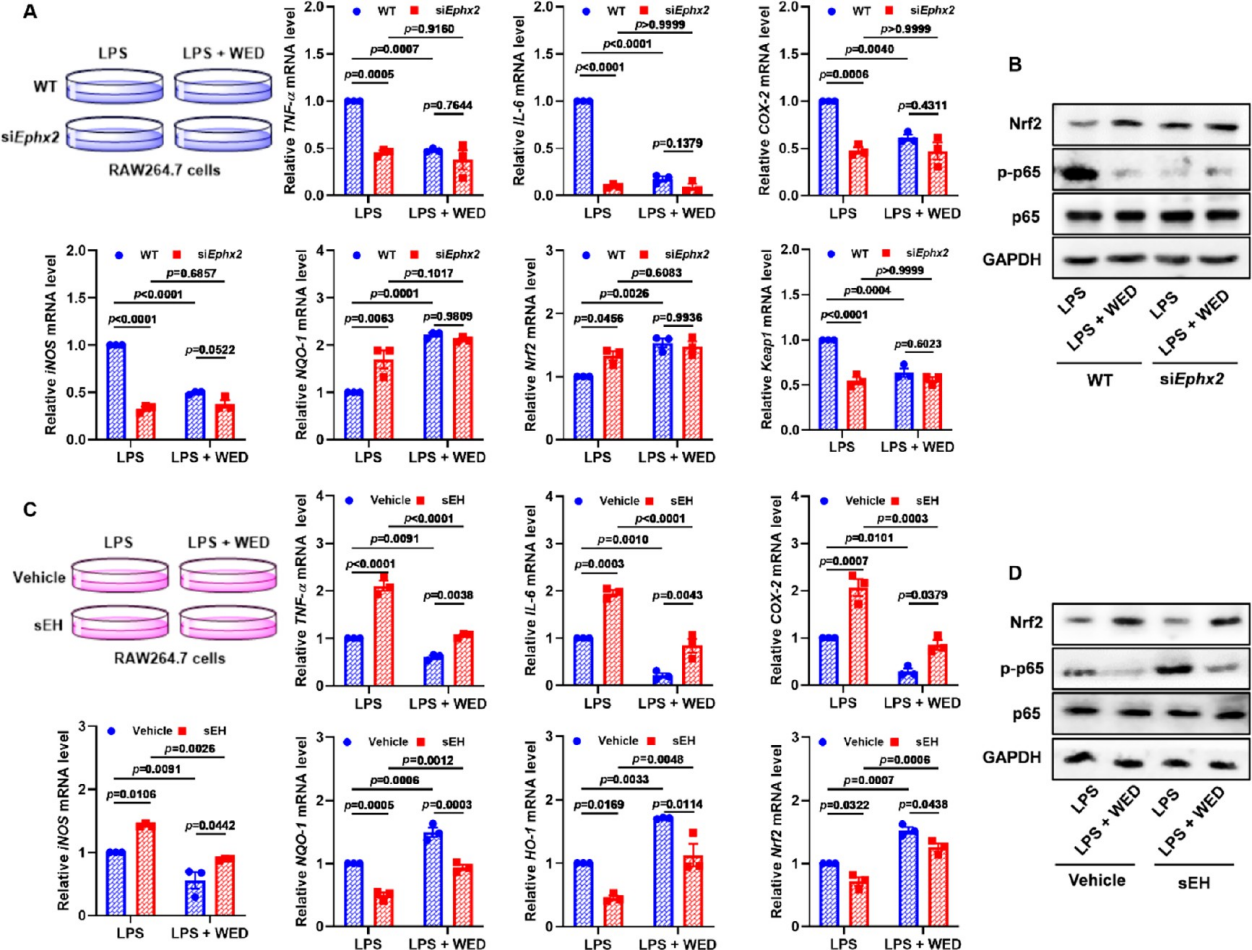
sEH knockdown and rescue abolished anti-inflammatory and antioxidant effects of WED *in vitro*. (A) sEH knockdown abolished the effects of WED toward inflammatory and antioxidant genes *TNF-α* (*p* = 0.0028; df = 1, 8; *F*-value = 18.0), *IL-6* (*p* < 0.0001; df = 1, 8; *F*-value = 355.1), *COX-2* (*p* = 0.0058; df = 1, 8; *F*-value = 13.9), *iNOS* (*p* < 0.0001; df = 1, 8; *F*-value = 133.8), *NQO-1* (*p* = 0.0037; df = 1, 8; *F*-value = 16.4), *Nrf2* (*p* = 0.0194; df = 1, 8; *F*-value = 8.5), and *Keap1* (*p* = 0.0006; df = 1, 8; *F*-value = 30.0) in LPS-induced RAW264.7 cells (mean ± SEM, *n* = 3, two-way ANOVA). (B) sEH knockdown abolished the effects of WED toward the NF-κB and Nrf2 pathways measured by Western blot. (C) sEH rescue weakened the effects of WED toward inflammatory and antioxidant genes *TNF-α* (*p* = 0.0006; df = 1, 8; *F*-value = 29.7), *IL-6* (*p* = 0.0921; df = 1, 8; *F*-value = 3.7), *COX-2* (*p* = 0.0489; df = 1, 8; *F*-value = 5.4), *iNOS* (*p* = 0.4830; df = 1, 8; *F*-value = 0.5), *NQO-1* (*p* = 0.6083; df = 1, 8; *F*-value = 0.3), *HO-1* (*p* = 0.8389; df = 1, 8; *F*-value = 0.04), and *Nrf2* (*p* = 0.8812; df = 1, 8; *F*-value = 0.02) in LPS-induced RAW264.7 cells (mean ± SEM, *n* = 3, two-way ANOVA). (D) sEH rescue weakened the effects of WED toward the NF-κB and Nrf2 pathways measured by Western blot.

Next, we performed sEH knockdown and rescue experiments to explore the role of sEH in anti-inflammatory and antioxidant effects of WED in LPS-stimulated macrophages. As shown in Figures S7 and S8, sEH knockdown and rescue could regulate the NF-κB and Nrf2 pathways, which are responsible for inflammatory response and oxidative stress, in macrophages. Moreover, sEH knockdown suppressed the increase of LPS-induced *TNF-α*, *IL-6*, *COX-2*, *iNOS*, and *Keap1* mRNA levels and reversed the decrease of LPS-induced *NQO-1* and *Nrf2* (Figure 6A,C). Knockdown also reversed effects of LPS toward transcript factors p65 and Nrf2 (Figure 6B), whereas the anti-inflammatory and antioxidant effects of WED were abolished in LPS-mediated sEH knockdown cells (Figures 6B and S10). In addition, sEH rescue aggravated LPS-induced inflammation and oxidative stress. This was illustrated by expressions of phosphorylated p65, Nrf2, and their downstream genes (Figures 6C,D and S10), while the protective effect of WED *in vitro* after sEH rescue was significantly weakened (Figures 6C,D and S10). These results demonstrate the effects of WED depend on its interaction with the sEH target.

### Phe362 and Gln384 Played Roles in the Binding of WED with sEHfacs

**Figure 7:**
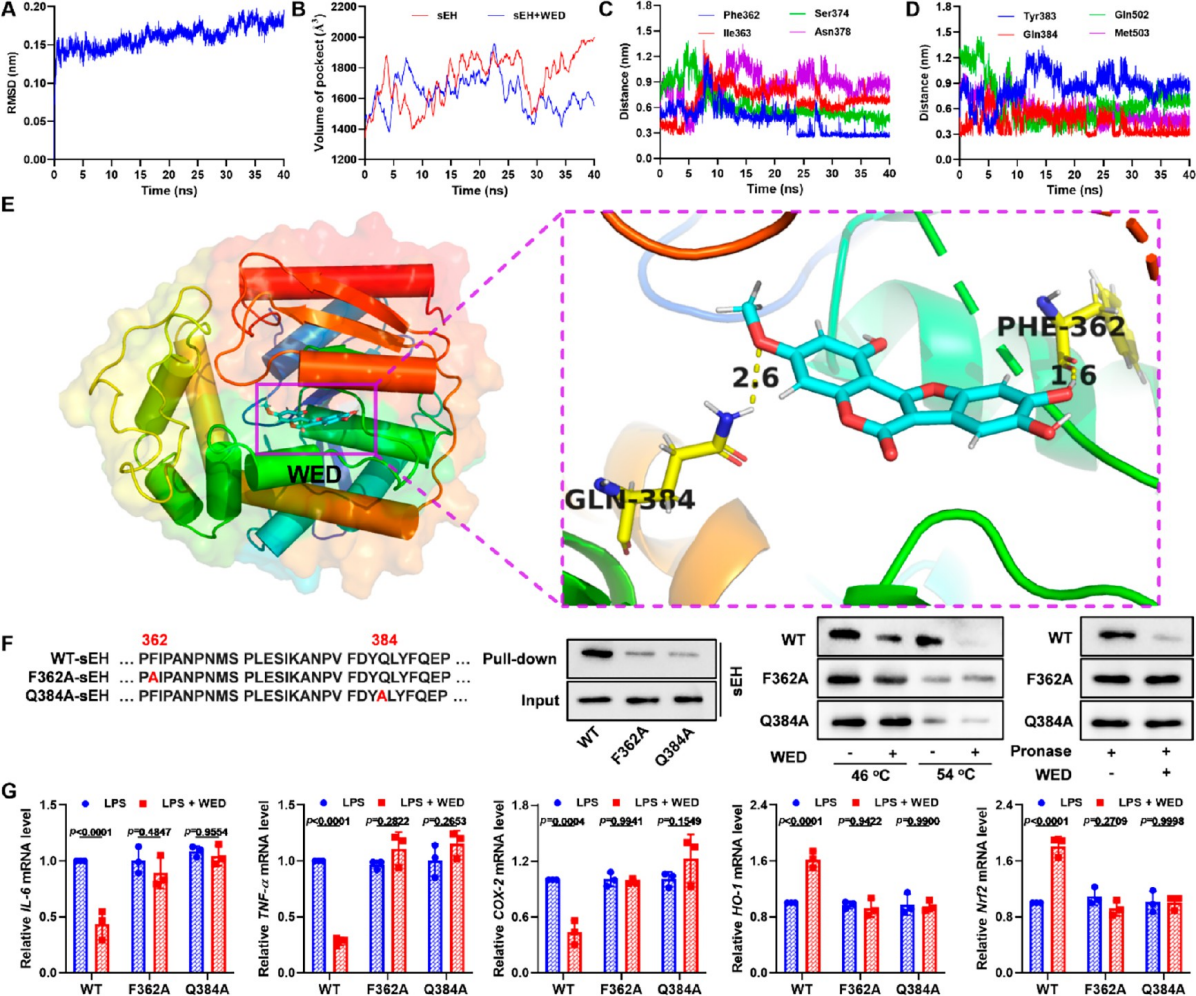
Phe362 and Gln384 played roles in the binding of WED with sEH. (A) The RMSD plot analyzed by molecular dynamics. (B) Effect of WED against the volume of the pocket. (C) The trajectories of Phe362, Ile363, Ser374, and Asn378 with WED. (D) The trajectories of Tyr363, Gln384, Gln502, and Met503 with WED. (E) The interactions of WED with sEH through hydrogen bonds of Phe362 and Gln384. (F) Phe362Ala and Gln384Ala mutations abolished the binding of WED with sEH. (G) Phe362Ala and Gln384Ala mutations abolished the anti-inflammatory [*IL-6*, *p* = 0.0014, df = (2, 12), *F*-value = 12.0; *TNF-α*, *p* < 0.0001, df = (2, 12), *F*-value = 37.4; *COX-2*, *p* = 0.0005, df = (2, 12), *F*-value = 15.1] and antioxidative effects [*HO-1*, *p* = 0.0001, df = (2, 12), *F*-value = 20.6; *Nrf2*, *p* < 0.0001, df = (2, 12), *F*-value = 30.5] of WED in LPS-stimulated RAW264.7 cells (mean ± SEM, *n* = 3, two-way ANOVA).

To explore the WED-binding sites on sEH, molecular dynamics stimulations were performed (Figures 7 and S11). The root-mean-square deviation (RMSD) of the WED–sEH complex remained about 1.5 Å (Figure 7A) with the binding energy of −108.51 kJ/mol (Figure S11) during the 40 ns of the molecular dynamic stimulation, revealing the stability of the WED–sEH complex. The protein trajectories of apo-sEH and the WED–sEH complex revealed that the binding of WED with sEH reduced the volume of the catalytic cavity of sEH (Figures 7B and S11E,F). There were still about 1–4 hydrogen bonds between WED and Phe362, Ile363, Ser374, Asn378, Tyr383, Gln384, Gln502, and Met503 in the molecular dynamic stimulation (Figure 7C,D). The result of 40 ns of molecular dynamic stimulation was plotted in Figure 7E, which shows that WED interacted with MD2 through two hydrogen bond interactions of amino acid residue Phe362 and Gln384 with distances of 1.6 and 2.6 Å, respectively, thereby demonstrating the role of Phe362 and Gln384 in the binding of WED with sEH. Next, we mutated Phe362 and Gln384 into Phe362Ala and Gln384Ala, respectively, for exploration of their roles. In the pull-down experiment based on the biotin–avidin system of the probe WED coupled with biotin (Bio-WED), the Phe362Ala and Gln384Ala mutations abolished the binding of WED with sEH (Figure 7F), and similar results were afforded from the experiments of CETSA and DARTS (Figure 7F). The Phe362 and Gln384 mutations abolished the anti-inflammatory and antioxidative effects of WED in LPS-stimulated RAW264.7 cells, as well (Figure 7G). These findings illustrate that amino acid residues Phe362 and Gln384 are important sites for WED to bind to sEH.

### GSK3β Was the Downstream Key Pathway of sEH in Anti-Inflammatory and Antioxidant Effects of WED

**Figure 8:**
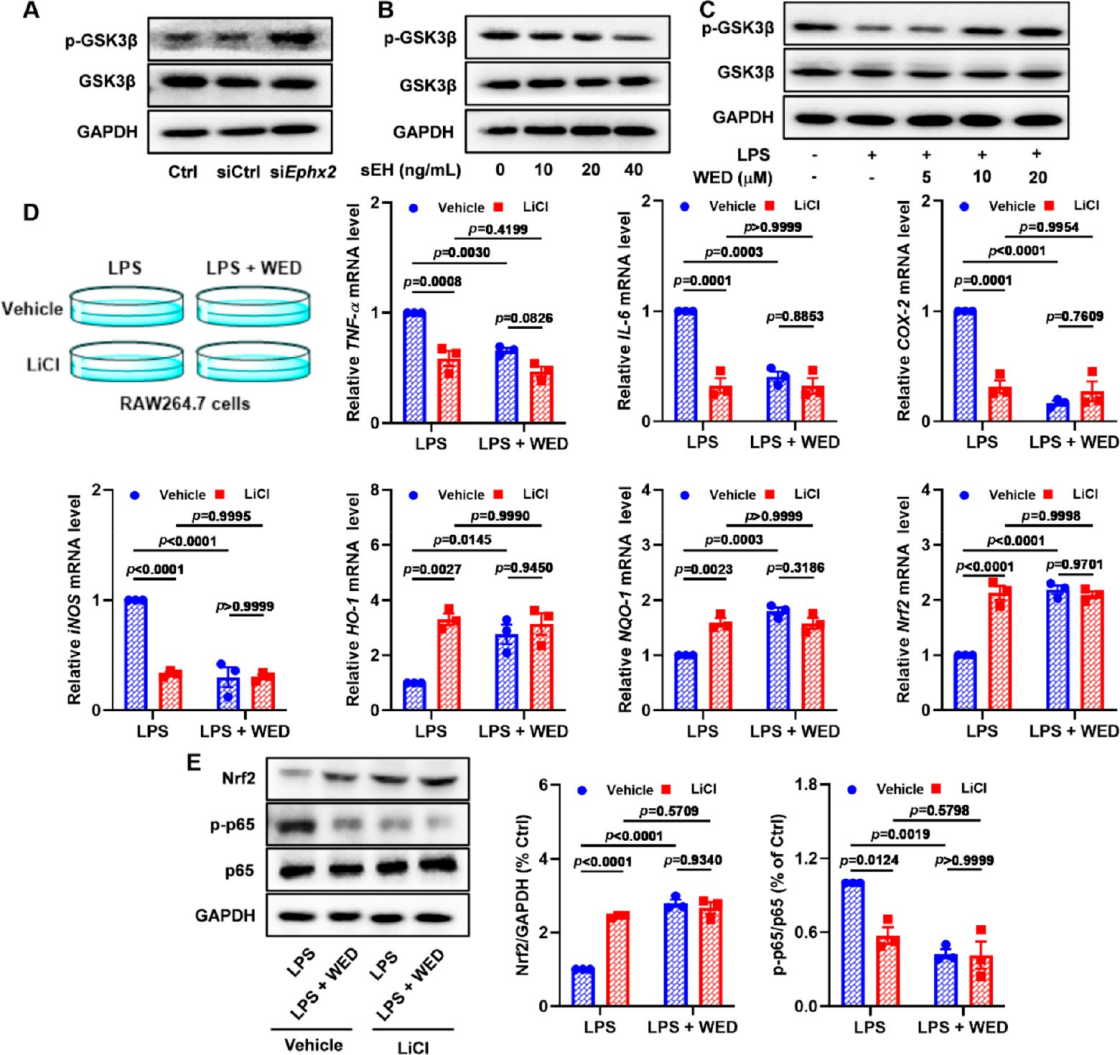
GSK3β is the downstream key pathway of sEH in the anti-inflammatory and antioxidant effects of WED. (A) sEH knockdown suppressed the GSK3β activity. (B) sEH rescue activated the GSK3β activity. (C) Inhibition of sEH by WED suppressed the GSK3β activity. (D) Inhibition of GSK3β by LiCl abolished effects of WED toward inflammatory and antioxidant genes *TNF-α* (*p* = 0.0316; df = 1, 8; *F*-value = 6.8), *IL-6* (*p* = 0.0006; df = 1, 8; *F*-value = 29.8), *COX-2* (*p* < 0.0001; df = 1, 8; *F*-value = 52.5), *iNOS* (*p* = 0.0001; df = 1, 8; *F*-value = 50.2), *HO-1* (*p* = 0.0097; df = 1, 8; *F*-value = 11.4), *NQO-1* (*p* = 0.0005; df = 1, 8; *F*-value = 32.2), and *Nrf2* (*p* < 0.0001; df = 1, 8; *F*-value = 55.7) *in vitro* (mean ± SEM, *n* = 3, two-way ANOVA). (E) Inhibition of GSK3β by LiCl abolished effects of WED toward the NF-κB (*p* = 0.0154; df = 1, 8; *F*-value = 9.4) and Nrf2 (*p* < 0.0001; df = 1, 8; *F*-value = 69.8) pathways measured by Western blot (mean ± SEM, *n* = 3, two-way ANOVA).

Accumulating evidence has demonstrated that the inhibition of sEH suppresses the GSK3β regulation of the NF-κB and Nrf2 pathways in the nervous system.^40^ First, we investigated the effect of sEH toward GSK3β in macrophages and found that sEH knockdown significantly promoted the phosphorylation of GSK3β (Ser9), thereby allowing the inhibition of the GSK3β activity (Figures 8A and S12A). Similarly, inhibition of sEH by WED could decrease the GSK3β activity in macrophages after LPS exposure (Figures 8C and S12C) while remarkably increasing the GSK3β activity after sEH rescue (Figures 8B and S12B). Therefore, we further investigated whether the downstream key pathway involving the sEH was involved in the protective effect of WED using the GSK3β inhibitor LiCl. Inhibition of GSK3β by LiCl (5 mM) attenuated macrophage-activation-mediated inflammation and oxidative stress, such as expressions of phosphorylated p65, Nrf2, and their downstream genes (Figure 8D,E). It is worth noting that the LiCl plus WED group did not display further additive or synergetic protective effects in inflammation and oxidative stress when compared with the WED group in LPS-induced macrophages (Figure 8D,E). Next, we investigated whether the inhibition of sEH regulated the GSK3β activity through EETs using the sEH substrate 14,15-EET (5 μM). Notably, 14,15-EET reduced the increase of LPS-induced GSK3β activity because of the upregulation of the phosphorylated-GSK3β level and abolished the effect of the inhibition of sEH by WED toward the GSK3β activity (Figure S13). These results suggest that the anti-inflammatory and antioxidant effects of WED depend on sEH to regulate the GSK3β-mediated NF-κB and Nrf2 pathways through EETs in macrophages.

### WED Inhibited the sEH Catalytic Activity and Led to the Inhibition of GSK3β *In Vivo*

**Figure 9:**
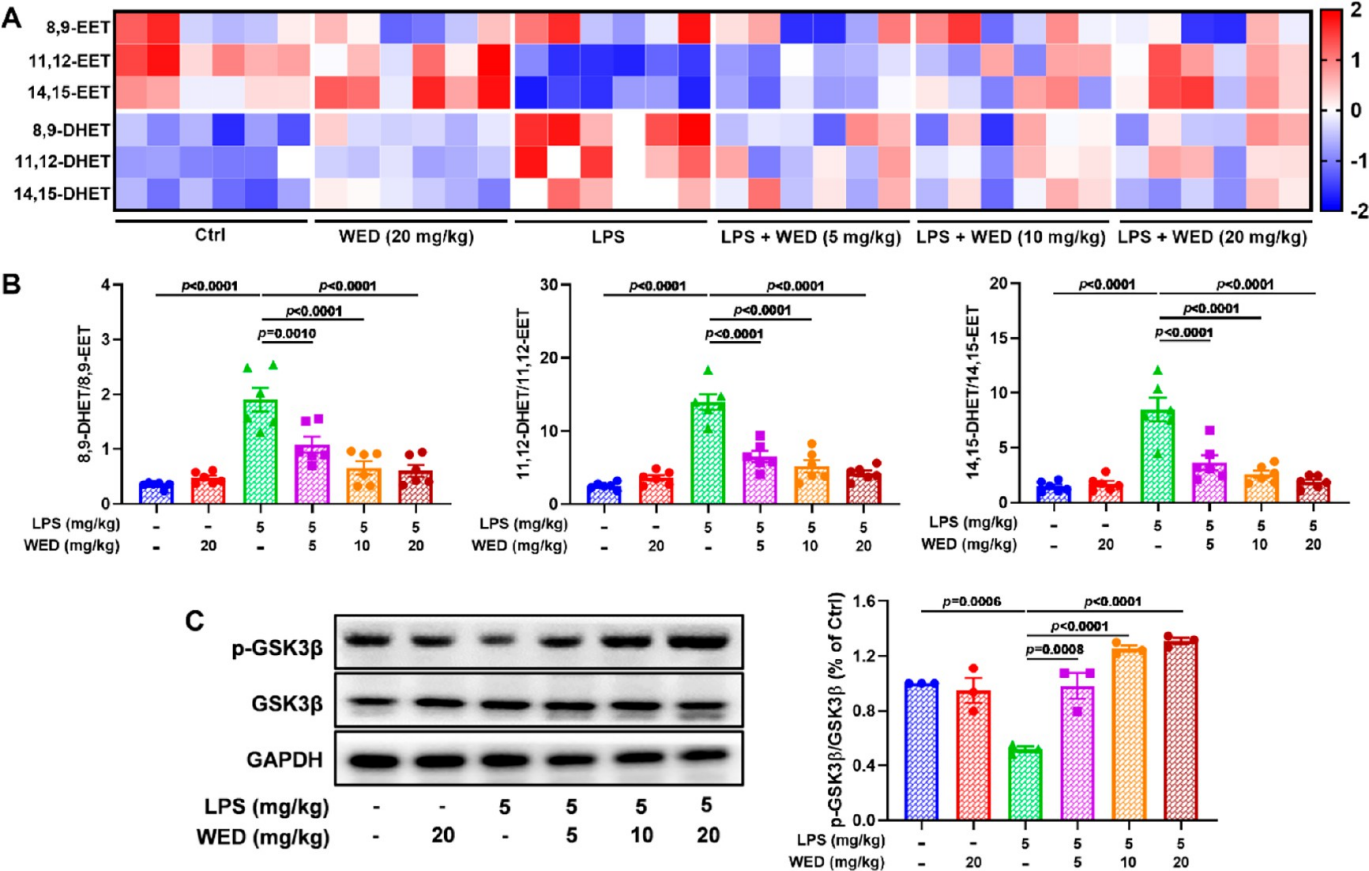
WED suppressed the sEH activity to allow the inhibition of GSK3β *in vivo*. (A) Heat map of sEH substrates (e.g., 8,9-EET, 11,12-EET, and 14,15-EET) and their corresponding diols (e.g., 8,9-DHET, 11,12-DHET, and 14,15-DHET). (B) WED reduced the ratio of 8,9-DHET/8,9-EET (*p* < 0.0001; df = 5, 30; *F*-value = 20.5), 11,12-DHET/11,12-EET (*p* < 0.0001; df = 5, 30; *F*-value = 37.5), and 14,15-DHET/14,15-EET (*p* < 0.0001; df = 5, 30; *F*-value = 22.9) *in vivo* to suppress the sEH activity (mean ± SEM, *n* = 6, one-way ANOVA). (C) Inhibition of sEH by WED led to the inactivation of the GSK3β activity (*p* < 0.0001; df = 5, 12; *F*-value = 25.3) in LPS-induced ALI mice (mean ± SEM, *n* = 3, one-way ANOVA).

In the LPS-induced ALI mouse model, we detected the sEH level and demonstrated the sEH overexpression in ALI mice (Figure S14). Next, we found that WED treatment significantly inhibited the decrease of levels of sEH substrates, such as 11,12-EET and 14,15-EET, except for in the case of low dose WED treatment (5 mg/kg, Figures 9A and S15). Conversely, the effect of WED toward 8,9-EET was not significant in LPS-exposed ALI mice except for the high dose of WED (20 mg/kg, Figures 9A and S15). As expected, administration of WED remarkably decreased levels of the corresponding diols, including 8,9-DHET, 11,12-DHET, and 14,15-DHET. Notably, WED treatment suppressed the sEH catalytic activity on the basis of the ratio of EETs and their corresponding DHETs (Figure 9B). Additionally, inhibition of sEH by WED treatment significantly suppressed the GSK3β pathway by promoting its phosphorylation at Ser9 (Figure 9C). All of the abovementioned results demonstrate the effect of sEH inhibition by WED on the GSK3β pathway in LPS-induced ALI mice.

### sEH Genetic KO Abolished the Pulmonary Protective Effect of WED

**Figure 10:**
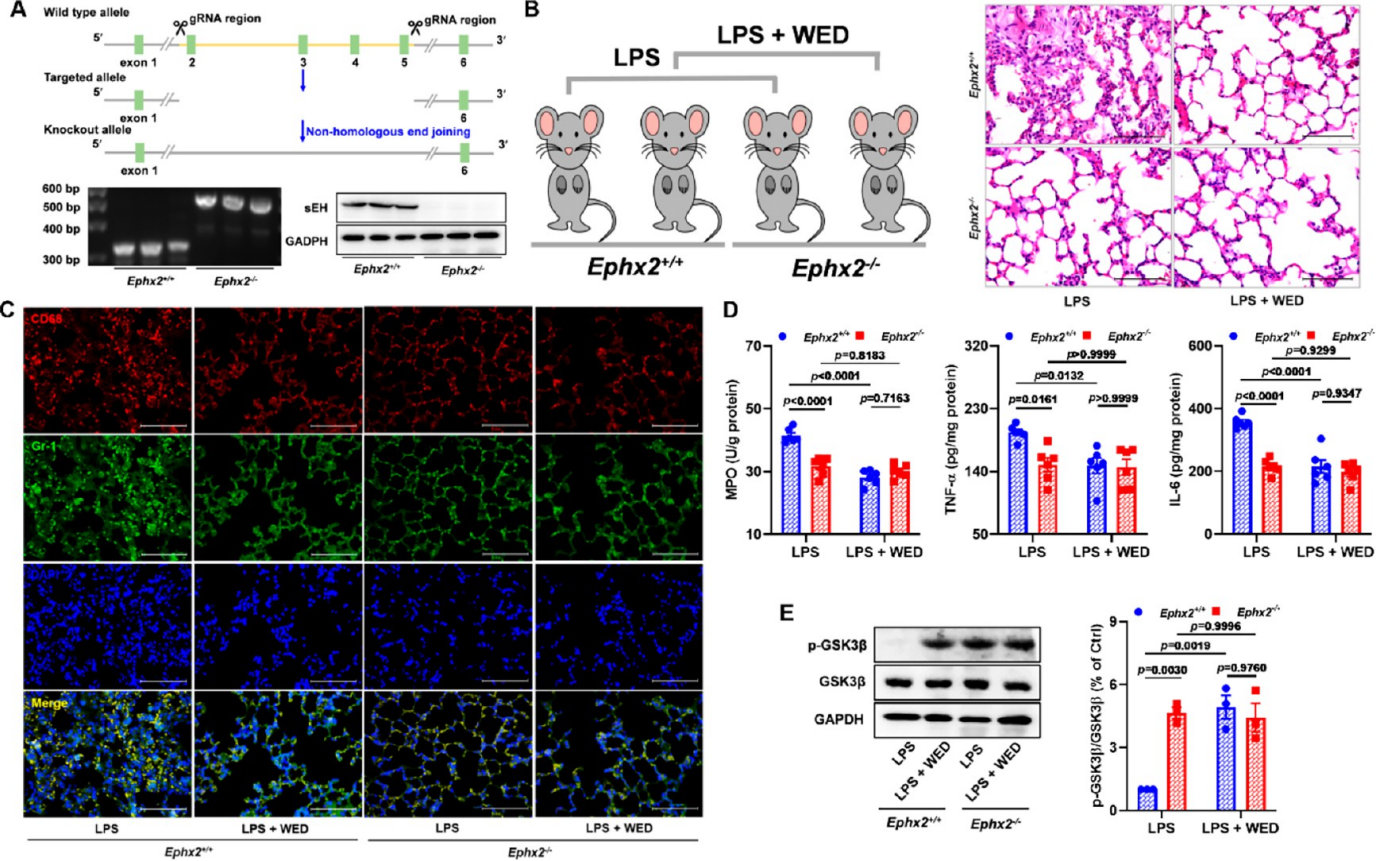
sEH genetic KO abolished the pulmonary protective effect of WED. (A) Genotyping and confirmation of sEH knockout mice. (B,C) Representative plots of H&E (B) and CD68 and Gr-1 (C) staining in LPS-induced ALI *Ephx2*^+/+^ and *Ephx2*^–/–^ mice treated with WED. (D) Measurement of pulmonary MPO (*p* < 0.0001; df = 1, 20; *F*-value = 32.7), TNF-α (*p* = 0.0323; df = 1, 20; *F*-value = 5.3), and IL-6 (*p* = 0.0002; df = 1, 20; *F*-value = 20.5) from LPS-induced ALI *Ephx2*^+/+^ and *Ephx2*^–/–^ mice treated with WED (mean ± SEM, *n* = 6, two-way ANOVA). (E) *Ephx2* KO abolished the effect of WED on the inhibition of GSK3β (*p* = 0.0019; df = 1, 8; *F*-value = 20.4) in LPS-induced ALI (mean ± SEM, *n* = 3, two-way ANOVA).

In order to explain whether the *in vivo* pulmonary protective effect of WED depended on the sEH target, we constructed sEH knockout (KO) mice through the deletion of four exons of sEH from exon 2 to exon 5, which were supported by the genotyping and Western blot analysis (Figure 10A). *Ephx2* genetic deletion attenuated pulmonary structural changes (Figure 10B), and the infiltration of macrophages and neutrophils (Figure 10C) contributed to a decrease in the MPO activity and levels of TNF-α and IL-6 in LPS-induced ALI (Figure 10D). Notably, the sEH KO plus WED group did not show further protective effects in LPS-induced ALI mice (Figure 10B–D). Meanwhile, suppression of sEH by WED via contribution to the GSK3β inhibition was not observed in LPS-mediated ALI *Ephx2*^*–/–*^ mice (Figure 10E). These results reveal that the pulmonary protective effect of WED depends on sEH.

### sEH Genetic KO Abolished Anti-Inflammatory and Antioxidant Effects of WED

**Figure 11:**
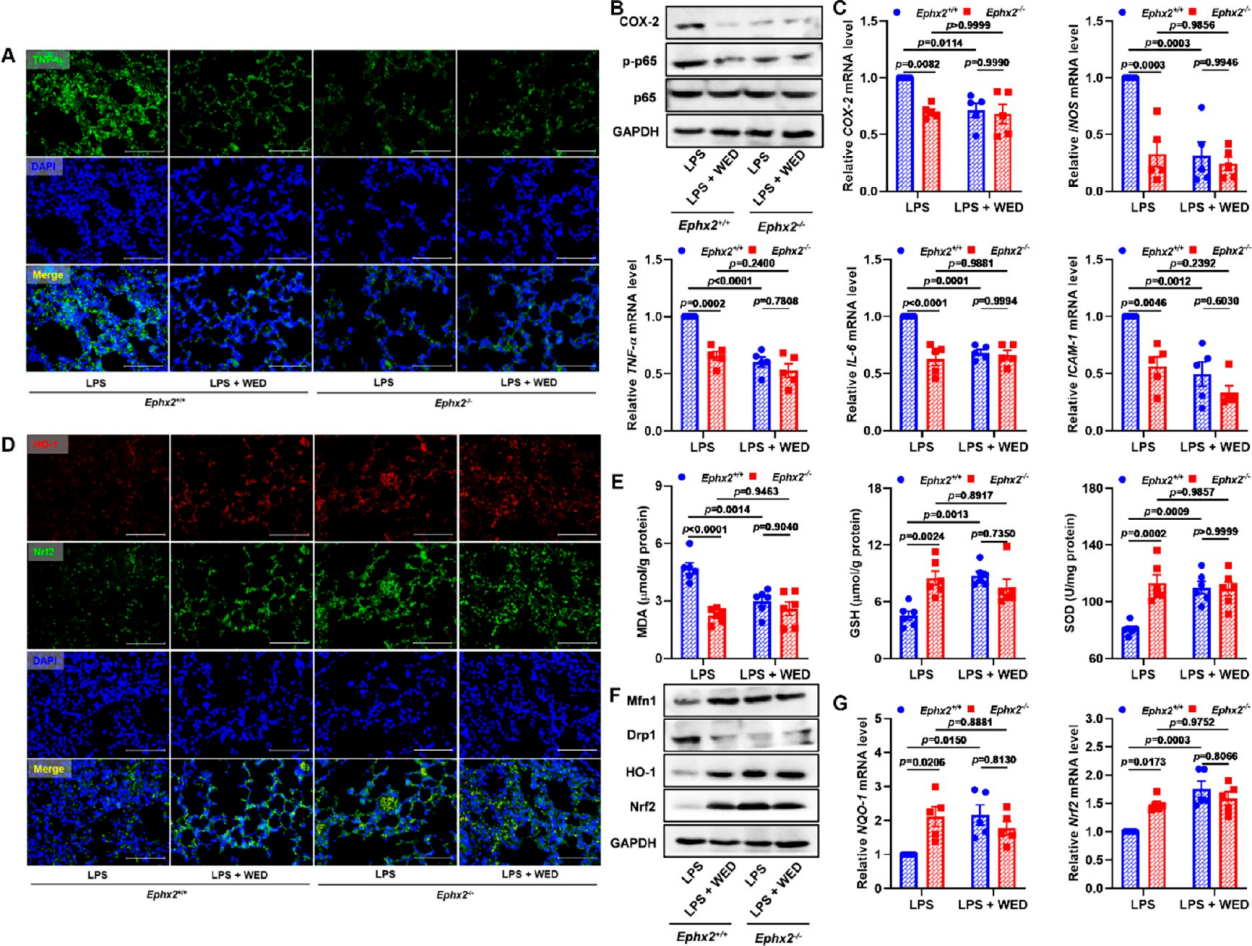
sEH genetic KO abolished the anti-inflammatory and antioxidant effects of WED. (A) Representative TNF-α staining plots in LPS-induced ALI *Ephx2*^+/+^ and *Ephx2*^–/–^mice treated with WED. (B) *Ephx2* KO abolished the effect of WED on expressions of COX-2 and p-p65/p65 in LPS-induced ALI. (C) *Ephx2* KO abolished the effect of WED on mRNA levels of *COX-2* (*p* = 0.0267; df = 1, 16; *F*-value = 6.0), *iNOS* (*p* = 0.0033; df = 1, 16; *F*-value = 11.9), *TNF-α* (*p* = 0.0058; df = 1, 16; *F*-value = 10.1), *IL-6* (*p* = 0.0003; df = 1, 16; *F*-value = 21.6), and *ICAM-1* (*p* = 0.0842; df = 1, 16; *F*-value = 3.4) in LPS-induced ALI (mean ± SEM, *n* = 5, two-way ANOVA). (D) Representative HO-1 and Nrf2 staining plots in LPS-induced ALI *Ephx2*^+/+^ and *Ephx2*^–/–^ mice treated with WED. (E) Measurement of pulmonary MDA (*p* = 0.0012; df = 1, 20; *F*-value = 14.3), GSH (*p* = 0.0008; df = 1, 20; *F*-value = 15.5), and SOD (*p* = 0.0012; df = 1, 20; *F*-value = 14.4) from LPS-induced ALI *Ephx2*^+/+^ and *Ephx2*^–/–^ mice treated with WED (mean ± SEM, *n* = 6, two-way ANOVA). (F) *Ephx2* KO abolished the effect of WED on expressions of Mfn1, Drp1, HO-1, and Nrf2 in LPS-induced ALI. (G) *Ephx2* KO abolished the effect of WED on mRNA levels of *NQO-1* (*p* = 0.0047; df = 1, 16; *F*-value = 10.8) and *Nrf2* (*p* = 0.0041; df = 1, 16; *F*-value = 11.2) in LPS-induced ALI (mean ± SEM, *n* = 5, two-way ANOVA).

Lastly, the effects of WED on LPS-induced inflammation and oxidative stress were investigated in *Ephx2*^+/+^ and *Ephx2*^–/–^mice. sEH genetic deletion led to a decrease in TNF-α positive cells (Figure 11A) and in levels of COX-2; phosphorylated p65; and the NF-κB target genes *COX-2*, *IL-6*, *iNOS*, *TNF-α*, and *ICAM-1* in comparison with the LPS-induced ALI *Ephx2*^+/+^ mice (Figures 11B,C and S16A). In addition, *Ephx2* genetic KO resulted in an increase in HO-1 and Nrf2 positive cells (Figure 11D) and in the levels of GSH and SOD (Figure 11E). The decreased level of MDA (Figure 11E) depended on the Nrf2 pathway, which contributed to the regulation of Mfn1, Drp1, Fis1, NQO-1, and Nrf2 (Figures 11F,G and S16B). Further effects were not observed in LPS-induced ALI *Ephx2*^–/–^ mice treated with WED. These results are consistent with anti-inflammatory and antioxidant effects of WED being dependent on sEH inhibition.

## Discussion

The C-terminal hydrolase activity of sEH is in a family of α/β hydrolase proteins. The inhibition of this C-terminal domain stabilizes levels of EETs to regulate various biological processes; therefore, sEH functions as a promising therapeutic target for diseases related to inflammation.^14,24^ In this study, sEH was identified as a key target for ALI by enhancing levels of EETs to suppress the macrophage activation. Moreover, we described the mechanistic insights into the sEH inhibition by the small natural molecule WED by targeting amino acid residues Phe362 and Gln384. Additionally, we demonstrated sEH as an important modulator of the GSK3β-mediated NF-κB and Nrf2 pathways in macrophages for inflammation and oxidative stress. These findings provided broader prospects for the treatment of ALI by targeting sEH to inhibit the macrophage activation and suggested WED as a potential agent for the development of sEH inhibitors.

Macrophages are cellular components of the innate immune system that reside in virtually all tissues and possess the capacity for cleaning apoptotic cells and releasing growth factors, thereby contributing to inflammation, innate immune response, and homeostasis.^2,3^ Depending on the pathogen recognition receptors, macrophages can phagocytize bacteria and other pathogens and inhaled particulates, especially in the respiratory system, meanwhile producing abundant inflammatory mediators,^1,41,42^ such as TNF-α and IL-6. This brings about mitochondria dysfunction via effects on the expression of proteins involved in the fusion and fission,^2,3,8^ such as Mfn1, Opa1, Drp1, and Fis1. Furthermore, the mitochondria release an amount of ROS, which contributes to oxidative stress through the inactivation of the Nrf2 pathway. Previous studies have demonstrated the effect of WED toward inflammation and oxidative stress in Parkinson’s disease and kidney injury.^33−37^ Furthermore, WED has attenuated bleomycin-mediated pulmonary fibrosis and protected bronchial epithelial cells from cigarette-smoke-extract-induced damage,^38,39^ which suggests the potential of WED against inflammation and oxidative stress in ALI. As our expected, these phenomena were all observed by regulating the NF-κB and Nrf2 pathways in LPS-induced macrophages and ALI animals after WED treatment.

Although the ability to define drug target protein recognition technology is rapidly developing, the discovery of drug targets still faces many challenges.^43^ Recent researches have reported the application of the affinity chromatography, biotin–avidin, and ligand-induced stability shift-dependent proteomics technologies for discovering the direct target of natural products, such as kurarinone, alisol B, eupalinolide B, and handelin.^24,44,45^ In this study, we uncovered that sEH was the direct cellular target of WED on the basis of technologies employing affinity chromatography. The sEH is a bifunctional enzyme with the C-terminal hydrolase and N-terminal phosphatase activities. The sEH is responsible for the hydrolysis of the bioactive metabolites EETs transformed by CYP2J, CYP2C, and other CYP enzymes from AA to their corresponding epoxides.^14,23^ Increasing evidence has revealed the role of sEH in inflammation and oxidative stress-mediated lung diseases.^46−48^ For example, chemical inhibition of sEH by 1-trifluoromethoxyphenyl-3-(1-propionylpiperidin-4-yl) urea (TPPU) suppressed LPS-mediated macrophage activation *in vitro* to regulate the proinflammatory cytokine level,^49−51^ increased the survival rate of LPS-induced ALI, and attenuated the neutrophil infiltration and alveolar capillary leakage in the ALI animal model.^49,50^ Furthermore, *Ephx2* genetic KO attenuated the pulmonary inflammation and edema in hyperoxia- or cigarette-smoke-mediated lung injury mice,^46−48^ thereby suggesting the potential of sEH in macrophage-activation-induced ALI. As expected, sEH knockdown alleviated the inflammation and oxidative stress, and the sEH rescue magnified the abovementioned changes *in vitro*. Similarly, sEH KO remarkably attenuated the course of ALI in an LPS-induced mouse model. Furthermore, the protective effect of WED was abolished by sEH knockdown, KO, and rescue.

Several sEH inhibitors, such as the ureas AR9281 (UC1153), EC5026 (a TPPU analog), and GSK2188931B (an amide), have entered human safety trials.^17,52^ They are all slow, tightly binding competitive inhibitors based on their urea or amide moieties because of their hydrogen bond interaction with amino acid residues Asp333, Tyr381, and Tyr465, in charge of opening and fixating on the epoxide ring, respectively.^17^ Recently, we have reported an uncompetitive sEH inhibitor kurarinone with an anti-PD effect and revealed that it interacted with Tyr343, Ile363, Gln384, and Asn472 in the surrounding of the catalytic cavity according to its cocrystal with sEH.^24^ Similarly, WED suppressed the sEH activity through hydrogen bonds with Phe362 and Gln384, which was supported by the results of the pull-down, CETSA, and DARTS experiments through Phe362Ala and Gln384Ala mutations.

Collectively, we first reported that sEH inhibition served as a target for the treatment of ALI through enhancing the EETs level to regulate GSK3β-mediated NF-κB and Nrf2 pathways, which resulted in the inactivation of macrophages *in vitro* and *in vivo*. Furthermore, we identified small molecule WED as an inhibitor for targeting sEH through the interaction with amino acid residues Phe362 and Gln384. These findings provid broader prospects for the ALI treatment by targeting sEH to alleviate the inflammation and oxidative stress and suggest WED as a natural product drug and a leading candidate for the development of new sEH inhibitors.

## Materials and Methods

### Chemicals and Reagents

WED was isolated and identified from *Inula britannica* on the basis of the ^1^H and ^13^C NMR spectra (Figures S17 and S18) with support of a check of purity by thin-layer chromatography and purity and structure by LC-MS. The primary antibodies for sEH (10833-1-AP), NQO-1 (A1518), GSK3β (A2081), p-GSK3β (5558), TNF-α (17590-1-AP), IL-6 (21865-1-AP), iNOS (13120), MCP-1 (25542-1-AP), COX-2 (12282), p65 (8242), p-p65 (3033), Mfn1 (13798-1-AP), Mfn2 (12186-1-AP), HO-1 (A19062), Drp1 (12957-1-AP), GCLC (12601-1-AP), Opa1 (27733-1-AP), GCLM (14241-1-AP), Fis1 (10956-1-AP), Nrf2 (16396-1-AP), and Keap1 (10503-2-AP) were purchased from Cell Signaling Technology (CST, Danvers, MA, USA), Abclonal (Wuhan, China), Proteintech (Wuhan, China), Abcam (Massachusetts, USA), and Affinity (Cincinnati, OH, USA). Recombinant human and mouse sEH afforded from Prof. Bruce D. Hammock (University of California) were gifts.

### Cell Culture and Treatment

RAW264.7 macrophages were seeded into 96-well plates and cultured in DMEM with 10% fetal bovine serum (FBS) at 37 °C in humidified air containing 5% CO_2_ at 37 °C. Overnight, cells were treated with WED. After the incubation for 24 h, the cells were collected for the cellular viability using the CCK-8 kit. The cells were seeded into the 96-well plate overnight and pretreated with WED (5, 10, or 20 μM) for 1 h before the challenge with LPS (500 ng/mL). After 24 h, the supernatants were collected and analyzed for the anti-inflammatory and antioxidant effects of WED.

For the sEH rescue experiment, cells were pretreated with sEH (5, 10, or 20 ng/mL) for 1 h, and incubated with WED (20 μM). After 24 h of LPS exposure, the cells were harvested for PCR and Western blot analyses.

For the inhibition experiment of GSK3β, cells were pretreated with the GSK3β inhibitor LiCl (5 mM) or 14,15-EET (5 μM) for 1 h before WED (20 μM) treatment. Then cells were administrated LPS for 24 h before being harvested for PCR and Western blot analyses.

### Transient Transfection

Small interfering RNA of *Ephx2* (si*Ephx2*) and negative control siRNA (siCtrl) were designed and synthesized by GenePharma (Shanghai, China). siCtrl (5 μL) and si*Ephx2* (5 μL) were transiently transfected into RAW264.7 cells using the transfection reagent (5 μL). After 6 h of transfection, fresh DMEM was added when replacing the medium, and the cells were continuously incubated for 36 h. The cells were harvested for both PCR and Western blot analyses to evaluate the efficiency of si*Ephx2* silencing. For the confirmatory experiment of targeting sEH with WED, wild-type (WT) and si*Ephx2* cells were pretreated with WED for 1 h before the LPS challenge (500 ng/mL). Cells were harvested for PCR and Western blot analyses after 24 h of incubation.

### ROS Detection by Flow Cytometry and Fluorescent Microscopic Analysis

Cells were pretreated with WED (5, 10, or 20 μM) for 1 h before the challenge with LPS. After 24 h, the ROS were measured by using probe DCFH-DA for 30 min at 37 °C. The cells were collected and used for the analysis of ROS-positive cells through the flow cytometry and a Leica DM4B microscope (Leica, Germany).

### Target Protein Fishing Assay

Epoxy-activated Sepharose 6B beads (GE Healthcare, Chicago, USA) coupled with WED were conducted according to the manufacturer’s protocol, as previously described.^44^ Cell lysates were incubated with WED beads or WED overnight at 4 °C, and PBS was used to elute the nonspecific binding proteins three times. The bead-captured proteins were analyzed by silver staining, Western blot, and a LC-MS/MS system.

WED coupled with biotin (Bio-WED) was synthesized and identified by NMR (Figures S19–S22), as previously described,^53^ for the colocalization of WED and sEH.

### Cellular Thermal Shift Assay (CETSA)

Cell lysates were incubated with WED (50 μM) for 30 min at 4 °C. The variable temperature experiments were performed at 46, 50, 54, 58, and 62 °C for 3 min, and the supernatants, afforded by the centrifugation, were analyzed by Western blot using the sEH antibody. The vehicle was used as the control group.

### Drug Affinity Responsive Target Stability (DARTS) Assay

Cell lysates pretreated with WED (5, 10, and 20 μM) for 1 h were treated with Pronase (7.5 μg/mL) and incubated for 15 min. After the centrifugation, the supernatants were analyzed by Western blot using the sEH antibody. The vehicle was used as the control group.

### Fluorescence-Based Binding Assay

WED (0.1 μM) was incubated with different concentrations of human recombinant sEH in PBS for 2 min, and then, the fluorescence signal was recorded on a microplate reader. The dissociation constant (*K*_d_) of WED with sEH was fitted as previously reported.^54^

### Microscale Thermophoresis (MST) Assay

First, using the Monolith NT kit to label the sEH protein,^55^ different concentrations of WED were added in the standard buffer with the labeled sEH protein (200 nM) for the incubation (15 min) and then analyzed on a Monolith NT.115 instrument (NanoTemper Technologies, München, Germany) at room temperature. All the data were analyzed by the NT analysis software to afford the *K*_d_ value of WED with sEH.

### Animal and Treatment

WT (*Ephx2*^+/+^*)* and sEH KO (*Ephx2*^–/–^) C57BL/6 mice (8 weeks, 22–24 g) were obtained from the Experimental Animal Center of Dalian Medical University (Dalian, China) and Cyagen Biosciences Inc. (Guangzhou, China), respectively, and kept under 12 h of light and 12 h of dark environment with a controlled temperature (22–24 °C) and humidity (50–60%).

First, WED (5, 10, or 20 mg/kg) and LPS (5 mg/kg) were dissolved in 10% hydroxypropyl β-cyclodextrin and saline, respectively, and then stored at 4 °C. Mice were randomly classified into six groups, including the control, WED (20 mg/kg), LPS, LPS + WED (5 mg/kg), LPS + WED (10 mg/kg), and LPS + WED (20 mg/kg) groups. WED (5, 10, or 20 mg/kg) was administrated intragastrically to mice in the WED and LPS + WED groups for a week by intragastric infusion, followed by administration of LPS (50 μL) through the intratracheal instillation after 1 h of the last administration of WED. Mice in the control and LPS groups were conducted with the corresponding vehicle or LPS (50 μL), according to the abovementioned protocol. After 24 h of LPS administration, BALF was collected as follows: the lungs were lavaged with 0.5 mL saline thrice, and the recovered fluid was used to analyze the levels of proteins IL-6 and TNF-α; activities of MPO and LDH; and the number of WBCs, PMNs, and MNs.

Second, *Ephx2*^+/+^ and *Ephx2*^–/–^ mice were classified into four groups (10 mice/group): the LPS (5 mg/kg)-treated *Ephx2*^+/+^ group, the LPS (5 mg/kg) + WED (20 mg/kg)-treated *Ephx2*^+/+^ group, the LPS (5 mg/kg)-treated *Ephx2*^–/–^ group, and the LPS (5 mg/kg) + WED (20 mg/kg)-treated *Ephx2*^–/–^ group. *Ephx2*^+/+^ and *Ephx2*^–/–^ mice were evaluated using the abovementioned protocol, and the lungs were collected.

### Pulmonary Pathological Assessments

After being fixed with 4% paraformaldehyde for 24 h and embedded with paraffin, the lungs were sliced into 4 μm thick sections, which were then used for the pulmonary pathological analysis according to the Hematoxylin and Eosin (H&E, Beyotime, Shanghai, China) kit.

### LC-MS/MS Analysis

The supernatant of the lung sample was collected after homogenization and centrifugation (20000 *g*) for 20 min at 4 °C, and then, levels of 8,9-EET, 11,12-EET, 14,15-EET, 8,9-DHET, 11,12-DHET, and 14,15-DHET were calculated on the basis of their standard curves and peak areas afforded from an AB Sciex Qtrap 5500 LC-MS/MS system (Foster City, CA, USA), respectively, as previously reported.^24^

### Immunohistochemical and Immunofluorescent Staining

For cell samples, cells were incubated with WED or the vehicle in 6-well plates before the LPS (500 ng/mL) challenge. After fixation, the cells were successively incubated with primary antibodies p65, Keap1, or Nrf2 at 4 °C overnight and the fluorescent secondary antibody for 1 h and then analyzed with a Leica DM4B microscope (Leica, Germany).

For the colocalization of WED with sEH, the cells were incubated with Bio-WED or the vehicle in 6-well plates for 1 h. After fixation, the cells were incubated with primary sEH antibody at 4 °C overnight and the fluorescent secondary antibody for 1 h and then analyzed by a Leica DM4B microscope (Leica, Germany).

For the lung samples, 10% goat serum was used for blocking microwaved lung sections for 20 min, followed by incubation with a cluster of CD68, Gr-1, TNF-α, HO-1, and Nrf2 antibodies overnight at 4 °C. The sections were washed with PBS three times before the incubation with normal or fluorescent secondary antibodies. Finally, the sections were used for the immunohistochemical and immunofluorescent analyses.

### Measurement of MPO, LDH, TNF-α, IL-6, MDA, GSH, and SOD

The concentrations of protein in cell, BALF, and lung samples were measured using the BCA method. The activities of MPO, LDH, and SOD and the contents of MDA and GSH in BALF and lung were measured using the appropriate kits (Jiancheng Bioengineering Institute, Nanjing, China). The levels of TNF-α and IL-6 were determined using their corresponding ELISA kits (Elabscience Biotechnology Co., Ltd., Wuhan, China).

### Real-Time Quantitative PCR

The total RNA in the cells and lungs was extracted with a TRIzol reagent, and its quantity and purity were analyzed in a Nanodrop spectrophotometer (Thermo, Waltham, MA, USA). The primers of *COX-2*, *Mfn1*, *Mfn2*, *Opa1*, *Drp1*, *MCP-1*, *ICAM-1*, *Fis1*, *HO-1*, *TNF-α*, *NQO-1*, *GCLC*, *IL-6*, *GCLM*, *Keap1*, *iNOS*, and *Nrf2* were added in the cDNA afforded after the reverse transcription, together with TransStart Tip Green qPCR SuperMix (Transgen, Beijing, China), respectively, and then real-time qPCR was performed on an Applied Biosystem 7500 Real-time PCR System (Thermo, Waltham, MA, USA). The copy number of every gene was normalized to the reference gene β-actin, and its relative mRNA expression was determined on the basis of the 2^–ΔΔCt^ method.

For the copy number of mtDNA, the DNA was determined using the Universal Genomic DNA Purification Mini Spin Kit (Beyotime, Shanghai, China) and assayed using the primers of mtDNA by real-time qPCR on the basis of the 2^–ΔΔCt^ method.

### Western Blot

Cell and lung samples were added in the lysis buffer with cocktail, homogenized, and centrifuged (15 000 rpm, 15 min, 4 °C) to obtain total proteins. Protein (20–30 μg) was loaded on 8%–12% SDS-PAGE for electrophoresis, and the polyvinylidene difluoride (PVDF) membranes transferred with the target protein were incubated with the corresponding primary antibodies overnight at 4 °C after blocking with 5% skim milk for 2 h. After the incubation with a horseradish peroxidase-conjugated secondary antibody, the membranes were incubated with the ECL reagent, followed by detection on the Tanon 5200-ECL detection system.

### Immunoprecipitation (IP)

Cell lysates were treated with WED (20 μM) at 4 °C for 30 min and then incubated with sEH or IgG antibody and protein A/G magnetic beads at 4 °C overnight. The resulting protein was analyzed by Western blot and the LC-MS/MS system.

### Soluble Epoxide Hydrolase Activity Assay

The inhibition of human and mouse sEH by WED was evaluated using PHOME as the substrate, as previously described.^24,56,57^

### Molecular Dynamics Stimulation

The interaction of WED with sEH (PDB: 4OCZ) was analyzed using the GROMACS package, according to previous methods,^24,56,57^ and plotted by PyMOL 2.4 software.

### Statistical Analysis

All the data are presented as means ± standard error of the mean (SEM) and analyzed on the basis of one-way ANOVA followed by Tukey’s test in Prism GraphPad Prism 8.0, except for the data in the experiments of sEH knockdown, sEH rescue, the inhibition of GSK3β by LiCl, and sEH KO experiment (two-way ANOVA followed by Sidak’s test). If the *p*-value was less than 0.05, the result was considered significant.

## Supporting Information Available

Supplementary Figures S1–22, including effects of WED, quantitative data of cells treated with WED, and spectra of WED and Bio-WED (PDF)
Transparent Peer Review report available (PDF)
