# Supplementary material for: Macrophage Inactivation by Small Molecule Wedelolactone via Targeting sEH for the Treatment of LPS-Induced Acute Lung Injury
Source: ACS Cent Sci. 2023 Feb 21;9(3):440–56. doi: 10.1021/acscentsci.2c01424 (PMC10037491; doi:10.1021/acscentsci.2c01424)
Supplement: Supplementary file 1 — oc2c01424_si_001.pdf [file oc2c01424_si_001.pdf]

Supplementary Materials for

Macrophage inactivation by small molecule wedelolactone via targeting sEH for the treatment of LPS-induced acute lung injury

Juan Zhang[a,b,c,+], Min Zhang[a,c,+], Xiao-Kui Huo[b,+], Jing Ning[a], Zhen-Long Yu[a], Christophe Morisseau[d], Cheng-Peng Sun[a,*], Bruce D. Hammock[d,*], Xiao-Chi Ma[b,*]

[a] College of Pharmacy, Dalian Medical University, Dalian 116044, China.

[b] Second Affiliated Hospital, Dalian Medical University, Dalian 116023, China

[c] School of Pharmaceutical Sciences, Health Science Center, Shenzhen University, Shenzhen 518061, China

[d] Department of Entomology and Nematology, UC Davis Comprehensive Cancer Center, University of California, Davis, CA 95616, United States

[+]These authors contributed equally to this work.

Corresponding authors at:

College of Pharmacy, Second Affiliated Hospital, Dalian Medical University, Dalian, China. E-mail: suncp146@163.com (C.P. Sun); maxc1978@163.com (X.C. Ma).

Department of Entomology and Nematology, UC Davis Comprehensive Cancer Center, University of California, Davis, United States. E-mail: bdhammock@ucdavis.edu (B.D. Hammock)

This file includes:

Supplementary Materials

Figure S1 Cytotoxicity of WED in RAW264.7 cells and the effect of WED on the release of NO in LPS-induced RAW264.7 cells

Figure S2 Effects of WED toward the mitochondrial fusion and fission *in vitro*.

Figure S3 Quantitative data of expressions of HO-1, NQO-1, GCLM, GCLC, Keap1, and Nrf2 in LPS-induced RAW264.7 cells treated with WED

Figure S4 Effects of WED toward inflammation and GSH in LPS-induced ALI mice

Figure S5 Effects of WED toward the mitochondrial fusion and fission in LPS-induced ALI mice.

Figure S6 Effect of WED toward oxidative stress in LPS-induced ALI mice

Figure S7 *Ephx2* knockdown inhibited inflammation and oxidative stress

Figure S8 sEH rescue promoted inflammation and oxidative stress.

Figure S9 The inhibitory effect of WED against mouse sEH

Figure S10 Quantitative data of p-p65/p65 and Nrf2 in LPS-stimulated *Ephx2* knockdown or sEH rescue cells treated with WED

Figure S11 Molecular dynamics analysis of WED and sEH

Figure S12 sEH regulated the GSK3β activity

Figure S13 14,15-EET abolished the effect of WED toward the GSK3β activity *in vitro*

Figure S14 Overexpression of sEH in ALI mice.

Figure S15 Effects of WED toward 8,9-EET, 11,12-EET, 14,15-EET, 8,9-DHET, 11,12-DHET, and 14,15-DHET LPS-induced ALI mice

Figure S16 *Ephx2* KO abolished effects of WED toward inflammation, oxidative stress, and mitochondria in LPS-induced ALI mice.

Figure S17 ^{1}H NMR (600 MHz, DMSO-d_6) spectrum of WED

Figure S18 ^{13}C NMR (150 MHz, DMSO-d_6) spectrum of WED

Figure S19 ^{1}H NMR (600 MHz, DMSO-d_6) spectrum of Bio-WED

Figure S20 ^{13}C NMR (150 MHz, DMSO-d_6) spectrum of Bio-WED

Figure S21 HSQC spectrum (600 MHz, DMSO-d_6) spectrum of Bio-WED

Figure S22 HMBC spectrum (600 MHz, DMSO-d_6) spectrum of Bio-WED

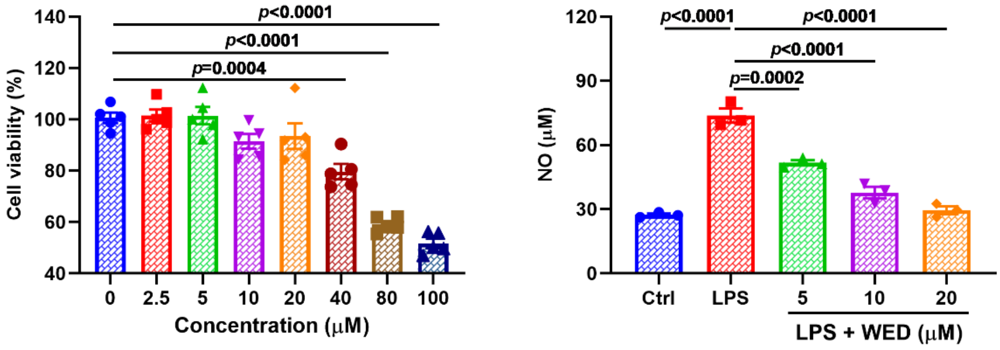

Figure S1 Cytotoxicity of WED in RAW264.7 cells (mean ± SEM, n = 5, one-way ANOVA, $p < 0.0001$, DF = 7, 32, F-value = 45.9) and the effect of WED on the release of NO in LPS-induced RAW264.7 cells (mean ± SEM, n = 3, one-way ANOVA, $p < 0.0001$, DF = 4, 10, F-value = 79.0).

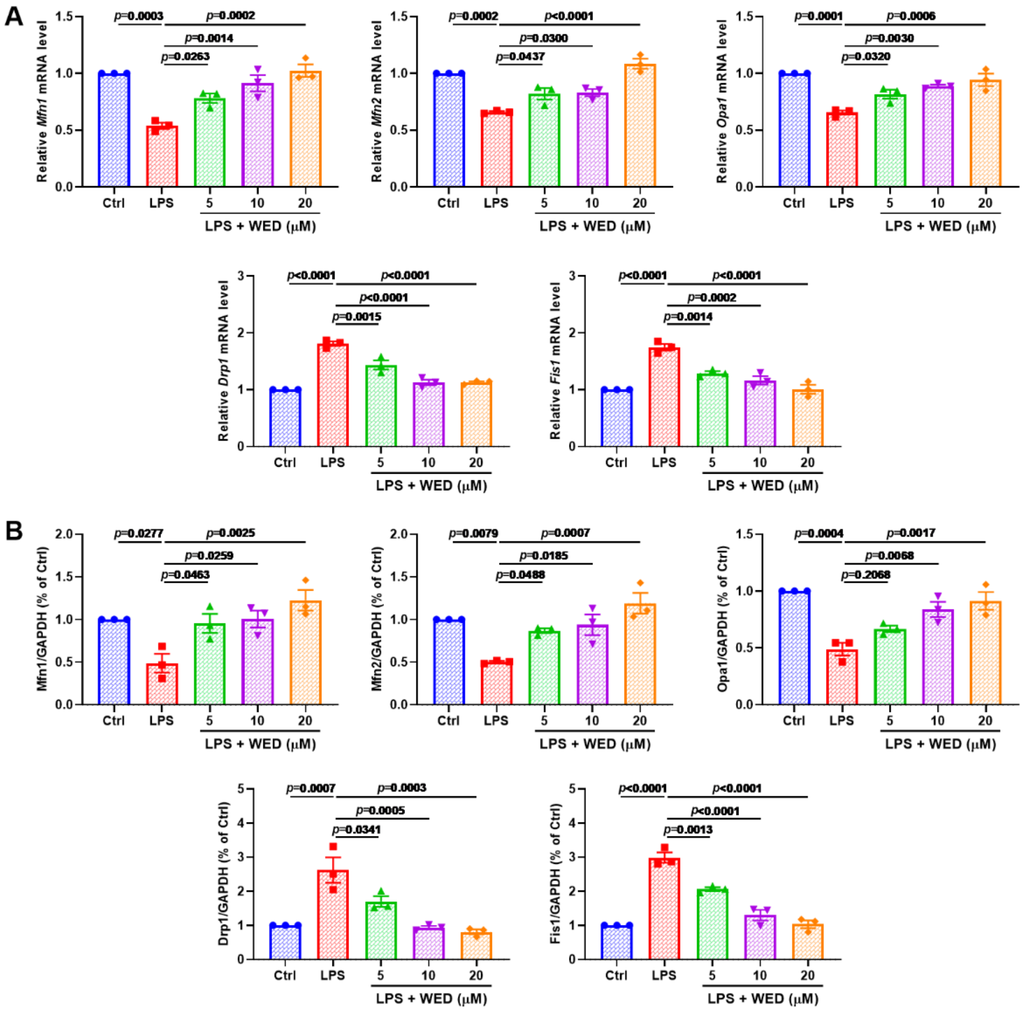

Figure S2 Effects of WED toward the mitochondrial fusion and fission *in vitro*. (A) WED regulated mRNA of genes *Mfn1* (p = 0.0001, DF = 4, 10, F-value = 18.5), *Mfn2* (p < 0.0001, DF = 4, 10, F-value = 24.9), *Op1* (p = 0.0002, DF = 4, 10, F-value = 17.4), *Drp1* (p < 0.0001, DF = 4, 10, F-value = 49.1), and *Fis1* (p < 0.0001, DF = 4, 10, F-value = 29.6) involved in the mitochondrial fusion and fission in LPS-induced RAW264.7 cells (mean ± SEM, n = 3, one-way ANOVA). (B) Quantitative data of expressions of Mfn1 (p = 0.0045, DF = 4, 10, F-value = 7.6), Mfn2 (p = 0.0014, DF = 4, 10, F-value = 10.4), Op1 (p = 0.0004, DF = 4, 10, F-value = 14.5), Drp1 (p = 0.0002, DF = 4, 10, F-value = 17.0), and Fis1 (p < 0.0001, DF = 4, 10, F-value = 55.8)

in LPS-induced RAW264.7 cells treated with WED (mean ± SEM, n = 3, one-way ANOVA).

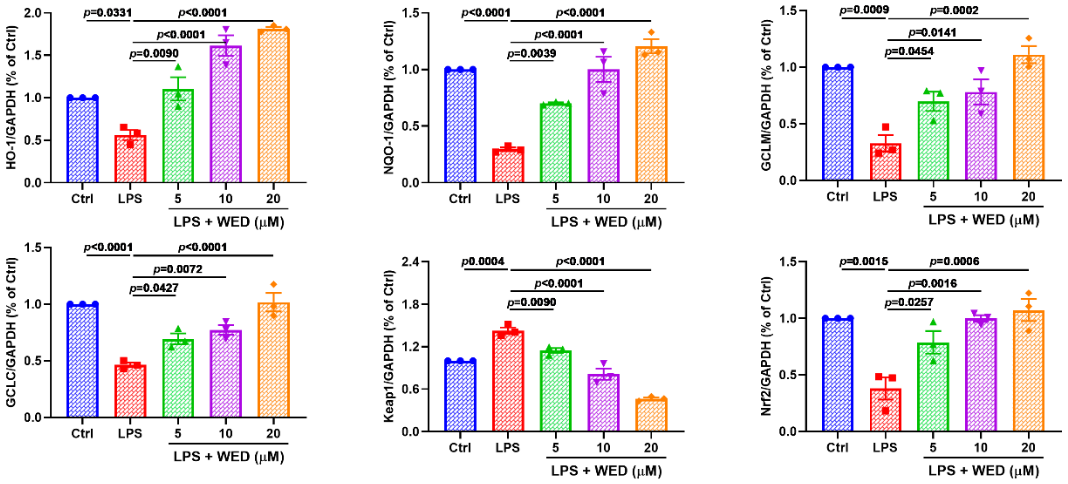

Figure S3 Quantitative data of expressions of HO-1 ($p < 0.0001$, DF = 4, 10, F-value = 33.0), NQO-1 ($p < 0.0001$, DF = 4, 10, F-value = 37.6), GCLM ($p = 0.0003$, DF = 4, 10, F-value = 15.2), GCLC ($p < 0.0001$, DF = 4, 10, F-value = 23.2), Keap1 ($p < 0.0001$, DF = 4, 10, F-value = 65.1), and Nrf2 ($p = 0.0005$, DF = 4, 10, F-value = 13.3) in LPS-induced RAW264.7 cells treated with WED (mean ± SEM, n = 3, one-way ANOVA).

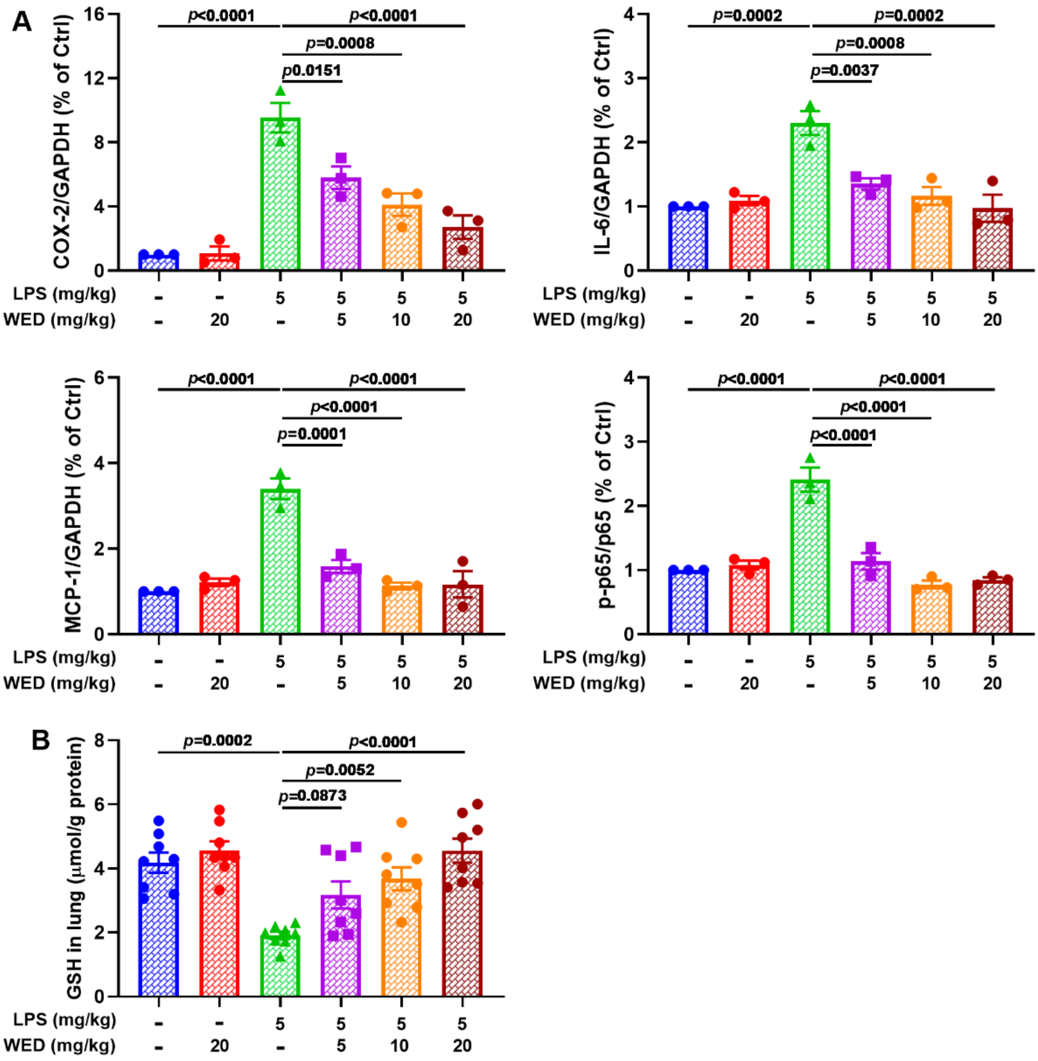

Figure S4 Effects of WED toward inflammation and GSH in LPS-induced ALI mice. (A) Quantitative data of expressions of COX-2 (p < 0.0001, DF = 5, 12, F-value = 25.1), IL-6 (p = 0.0001, DF = 5, 12, F-value = 13.6), MCP-1 (p < 0.0001, DF = 5, 12, F-value = 26.5), and p-p65/p65 (p < 0.0001, DF = 5, 12, F-value = 35.5) in LPS-induced ALI mice treated with WED (mean ± SEM, n = 3, one-way ANOVA). (B) The level of and GSH (p < 0.0001, DF = 5, 42, F-value = 9.8) in LPS-induced ALI mice treated with WED (mean ± SEM, n = 8, one-way ANOVA).

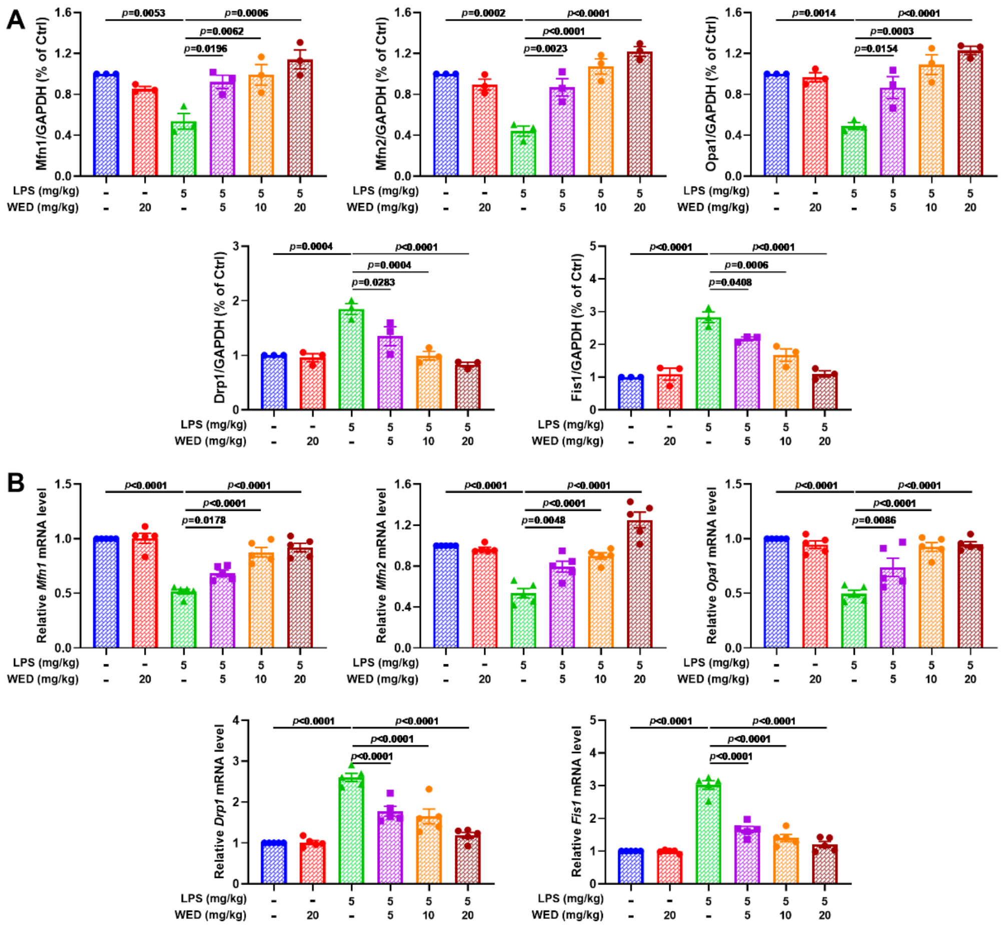

Figure S5 Effect of WED toward the mitochondrial fusion and fission in LPS-induced ALI mice. (A) Quantitative data of expressions of Mfn1 (p = 0.0011, DF = 5, 12, F-value = 8.7), Mfn2 (p < 0.0001, DF = 5, 12, F-value = 20.9), Op1 (p < 0.0001, DF = 5, 12, F-value = 9.8), Drp1 (p < 0.0001, DF = 5, 12, F-value = 15.7), and Fis1 (p < 0.0001, DF = 5, 12, F-value = 30.2) in LPS-induced ALI mice treated with WED (mean ± SEM, n = 3, one-way ANOVA). (B) WED regulated mRNA of genes *Mfn1* (p < 0.0001, DF = 5, 24, F-value = 33.2), *Mfn2* (p < 0.0001, DF = 5, 24, F-value = 28.3), *Op1* (p < 0.0001, DF = 5, 24, F-value = 19.0), *Drp1* (p < 0.0001, DF = 5, 24, F-value = 35.5), and *Fis1* (p < 0.0001, DF = 5, 24, F-value = 75.9) involved in the mitochondrial fusion and fission in LPS-induced ALI mice (mean ± SEM, n = 5,

one-way ANOVA).

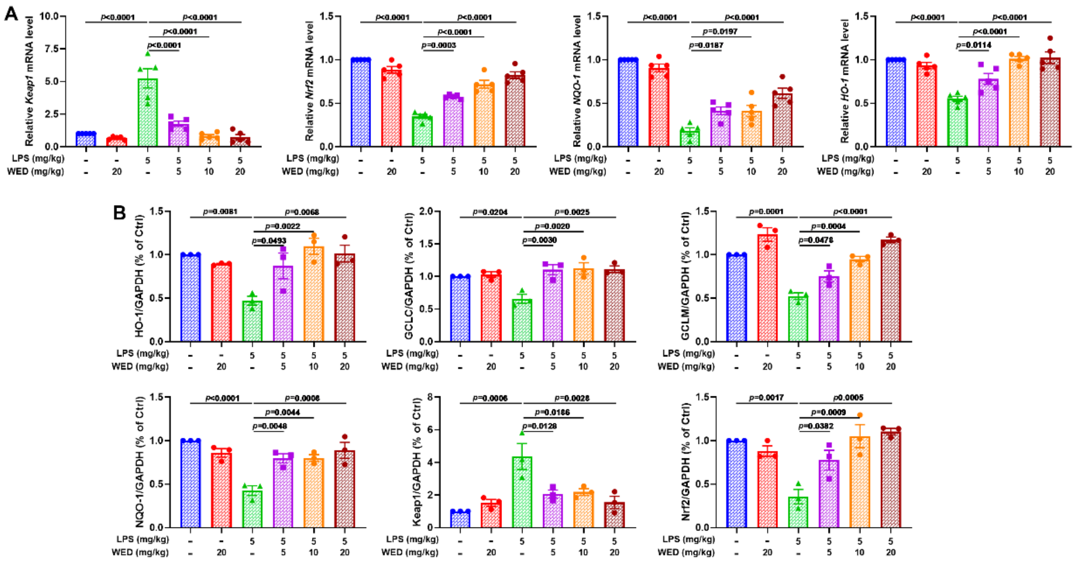

Figure S6 Effect of WED toward oxidative stress in LPS-induced ALI mice. (A) WED regulated mRNA of genes *Keap1* ($p < 0.0001$, DF = 5, 24, F-value = 30.2), *Nrf2* ($p < 0.0001$, DF = 5, 24, F-value = 59.0), *NQO-1* ($p < 0.0001$, DF = 5, 24, F-value = 46.5), and *HO-1* ($p < 0.0001$, DF = 5, 24, F-value = 18.7) in LPS-induced ALI mice (mean ± SEM, n = 5, one-way ANOVA). (B) Quantitative data of expressions of HO-1 ($p = 0.0001$, DF = 5, 12, F-value = 6.9), GCLC ($p = 0.0015$, DF = 5, 12, F-value = 8.1), GCLM ($p < 0.0001$, DF = 5, 12, F-value = 31.3), NQO-1 ($p = 0.0002$, DF = 5, 12, F-value = 12.6), Keap1 ($p = 0.0001$, DF = 5, 12, F-value = 9.1), and Nrf2 ($p = 0.0004$, DF = 5, 12, F-value = 10.6) in LPS-induced ALI mice treated with WED (mean ± SEM, n = 3, one-way ANOVA).

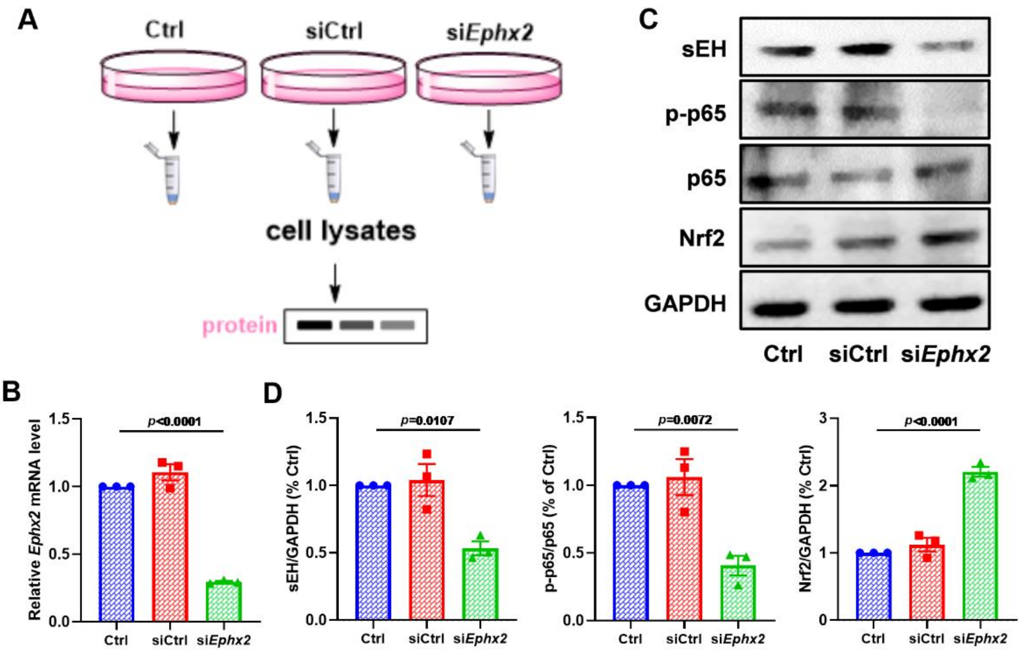

Figure S7 *Ephx2* knockdown inhibited inflammation and oxidative stress. (A) The protocol of *Ephx2* knockdown in RAW264.7 cells. (B) mRNA level of *Ephx2* (p < 0.0001, DF = 2, 6, F-value = 161.4) after *Ephx2* knockdown (mean ± SEM, n = 3, one-way ANOVA). (C) Expressions of sEH, p-p65, p65, and Nrf2 after *Ephx2* knockdown. (D) Quantitative data of sEH (p = 0.0052, DF = 2, 6, F-value = 14.2), p-p65/p65 (p = 0.0034, DF = 2, 6, F-value = 17.0), and Nrf2 (p < 0.0001, DF = 2, 6, F-value = 87.4) after *Ephx2* knockdown (mean ± SEM, n = 3, one-way ANOVA).

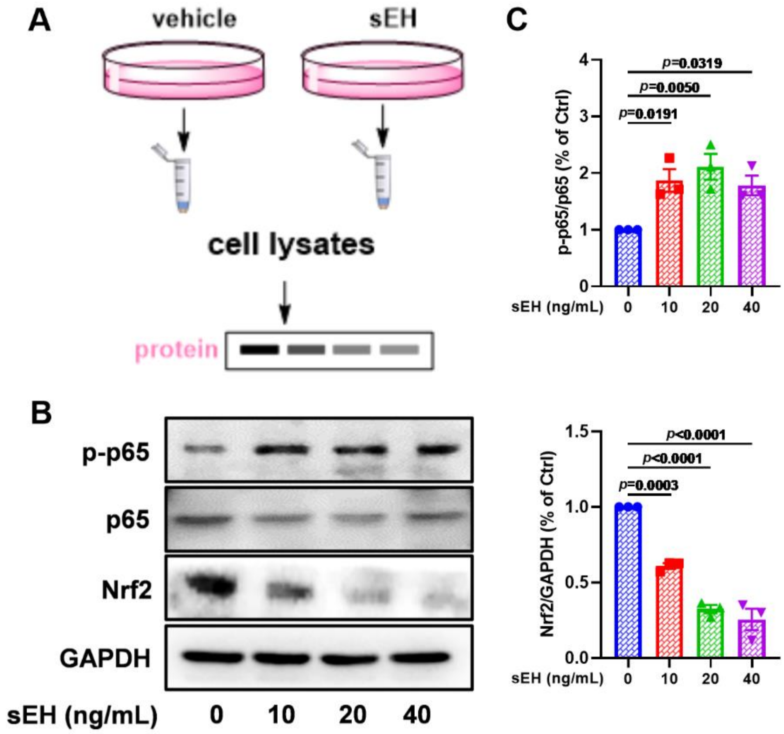

Figure S8 sEH rescue promoted inflammation and oxidative stress. (A) The protocol of sEH rescue in RAW264.7 cells. (B) Expressions of p-p65, p65, and Nrf2 after sEH rescue. (C) Quantitative data of p-p65/p65 (p = 0.0097, DF = 3, 8, F-value = 7.7) and Nrf2 (p < 0.0001, DF = 3, 8, F-value = 74.9) after sEH rescue (mean ± SEM, n = 3, one-way ANOVA).

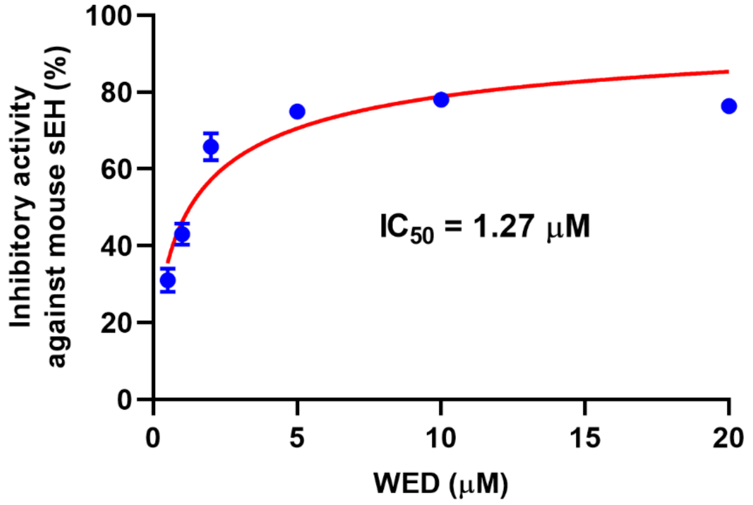

Figure S9 The inhibitory effect of WED against mouse sEH (mean ± SEM, n = 3).

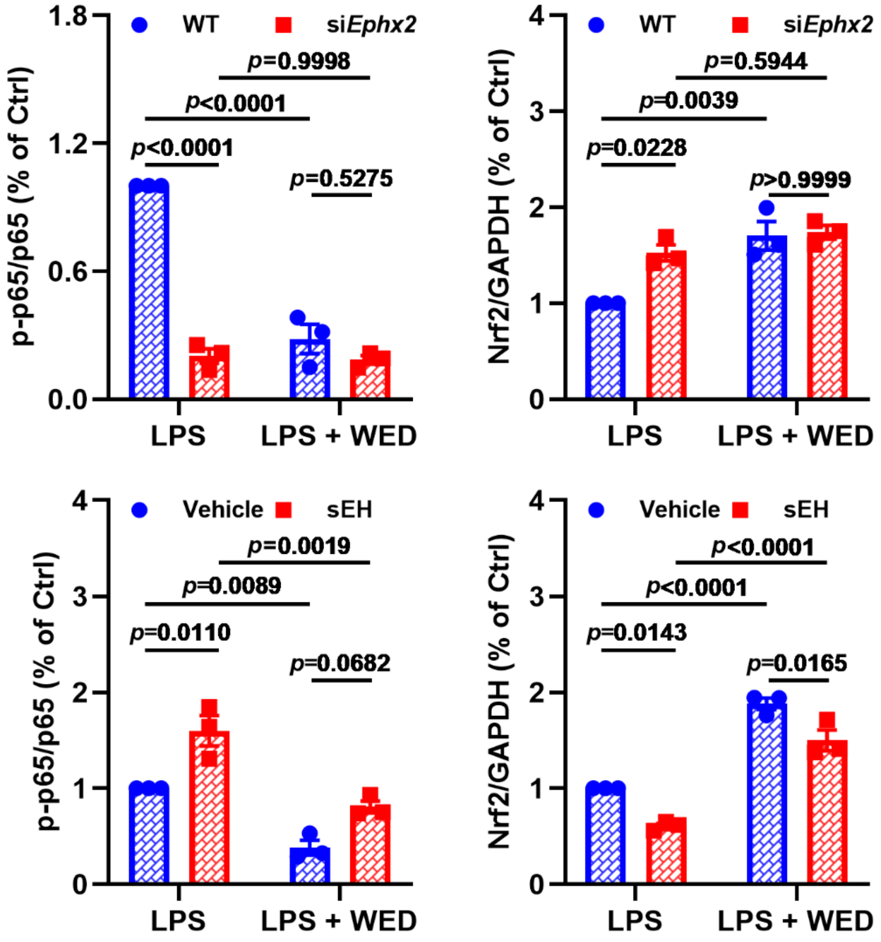

Figure S10 Quantitative data of p-p65/p65 (p < 0.0001, DF = 1, 8, F-value = 77.7; p = 0.3805, DF = 1, 8, F-value = 0.9) and Nrf2 (p = 0.0294, DF = 1, 8, F-value =7.0; p = 0.9422, DF = 1, 8, F-value = 0.006) in LPS-stimulated *Ephx2* knockdown or sEH rescue cells treated with WED (mean ± SEM, n = 3, one-way ANOVA).

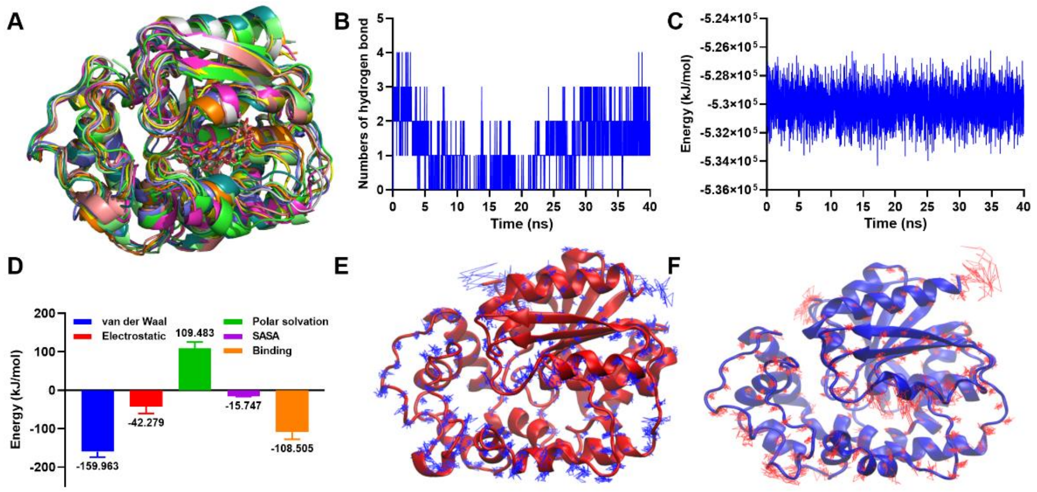

Figure S11 Molecular dynamics analysis of WED and sEH. (A) Overlapped plot of 3D structure of WED and sEH analyzed by molecular dynamics. (B) The number of hydrogen bonds in 40 ns of molecular dynamics stimulation. (C) The energy of the complex of WED and sEH. (D) The binding energy of WED with sEH. (E) Protein trajectories of sEH in 40 ns of molecular dynamics stimulation. (F) Protein trajectories of the complex of WED and sEH and in 40 ns of molecular dynamics stimulation.

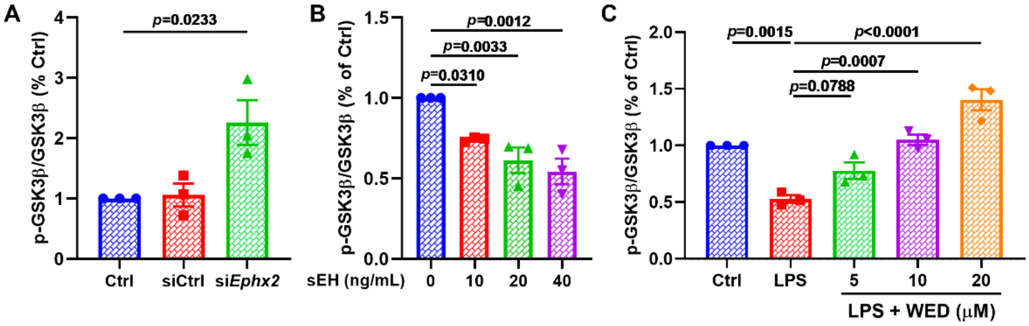

Figure S12 sEH regulated the GSK3β activity. (A) Quantitative data of the expression level of p-GSK3β/GSK3β (p = 0.0166, DF = 2, 6, F-value = 8.8) after *Ephx2* knockdown (mean ± SEM, n = 3, one-way ANOVA). (B) Quantitative data of the expression level of p-GSK3β/GSK3β (p = 0.0021, DF = 3, 8, F-value = 12.7) after sEH rescue (mean ± SEM, n = 3, one-way ANOVA). (C) Quantitative data of the expression level of p-GSK3β/GSK3β (p < 0.0001, DF = 4, 10, F-value = 30.9) in LPS-induced RAW264.7 cells treated with WED (mean ± SEM, n = 3, one-way ANOVA).

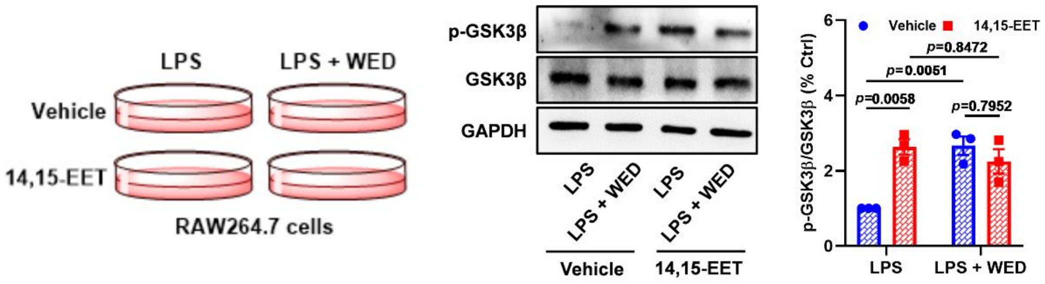

Figure S13 14,15-EET abolished the effect of WED toward the GSK3β activity *in vitro* (p = 0.0020, DF = 1, 8, F-value = 20.3).

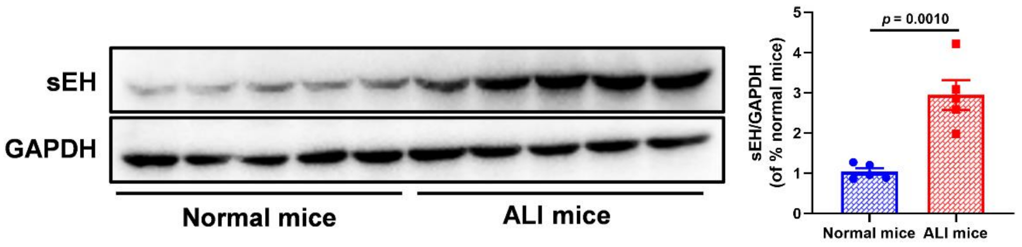

Figure S14 Overexpression of sEH in ALI mice (mean ± SEM, n = 5, *t*-test, p = 0.0140, DF = 4, 4, F-value =19.3).

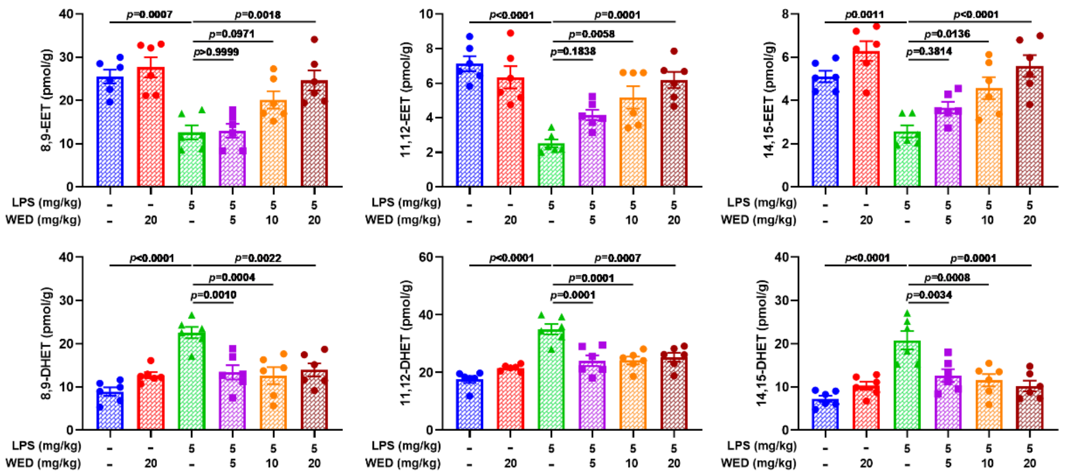

Figure S15 Effects of WED toward 8,9-EET (p < 0.0001, DF = 5, 30, F-value = 11.3), 11,12-EET (p < 0.0001, DF = 5, 30, F-value = 12.4), 14,15-EET (p < 0.0001, DF = 5,

30, F-value = 11.6), 8,9-DHET ($p < 0.0001$, DF = 5, 30, F-value = 10.2), 11,12-DHET ($p < 0.0001$, DF = 5, 30, F-value = 15.9), and 14,15-DHET ($p < 0.0001$, DF = 5, 30, F-value = 10.9) LPS-induced ALI mice (mean ± SEM, n = 6, one-way ANOVA).

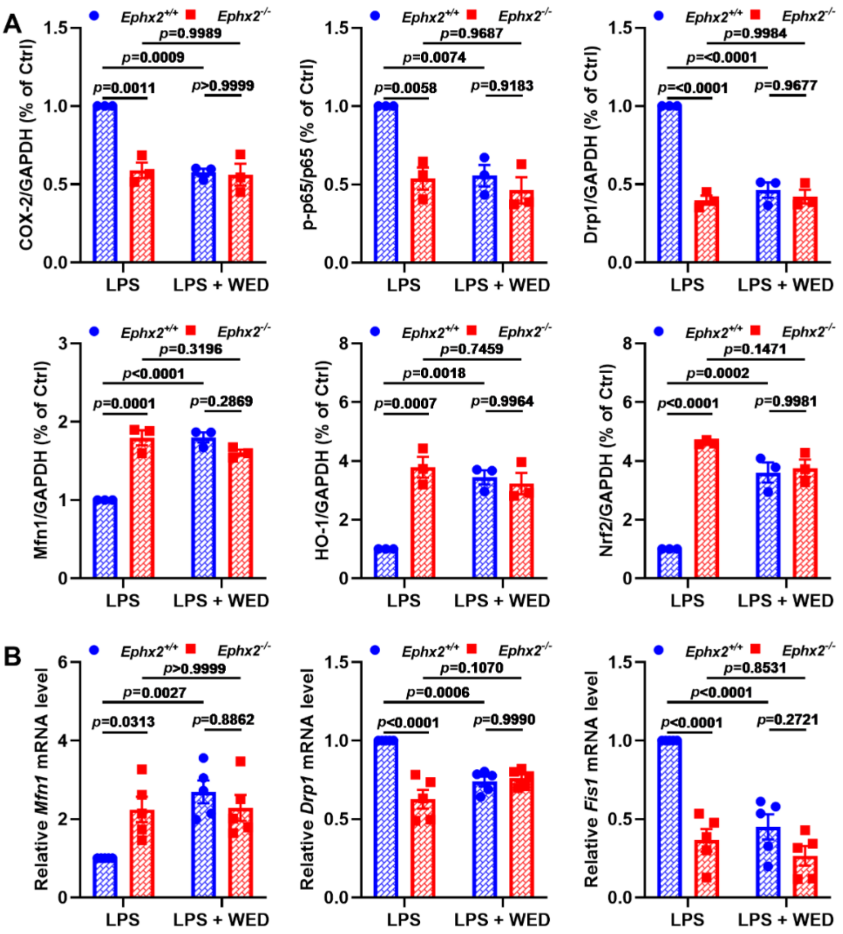

Figure S16 *Ephx2* KO abolished effects of WED toward inflammation, oxidative stress, and mitochondria in LPS-induced ALI mice. (A) Quantitative data of expressions of COX-2 ($p = 0.0021$, DF = 1, 8, F-value = 19.8), p-p65/p65 ($p = 0.0210$, DF = 1, 8, F-value = 8.2), Drp1 ($p < 0.0001$, DF = 1, 8, F-value = 61.2), Mfn1 ($p < 0.0001$, DF = 1, 8, F-value = 64.7), HO-1 ($p = 0.0007$, DF = 1, 8, F-value = 27.9), and

Nrf2 ($p < 0.0001$, DF = 1, 8, F-value = 59.1) in LPS-induced ALI *Ephx2*$^{+/+}$ and *Ephx2*$^{-/-}$ mice treated with WED (mean ± SEM, n = 3, two-way ANOVA). (B) *Ephx2* KO abolished the effect of WED on mRNA levels of *Mfn1* ($p = 0.0079$, DF = 1, 16, F-value = 9.2), *Drp1* ($p < 0.0001$, DF = 1, 16, F-value = 30.0), and *Fis1* ($p = 0.0023$, DF = 1, 16, F-value = 13.1) in LPS-induced ALI (mean ± SEM, n = 5, two-way ANOVA)

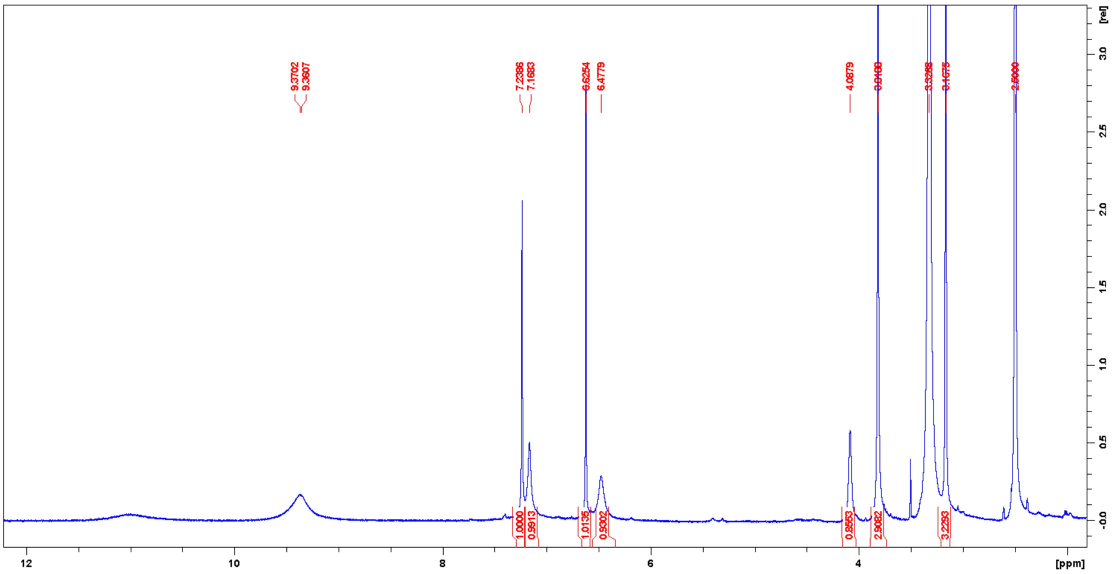

Figure S17 ^{1}H NMR (600 MHz, DMSO-d_6) spectrum of WED

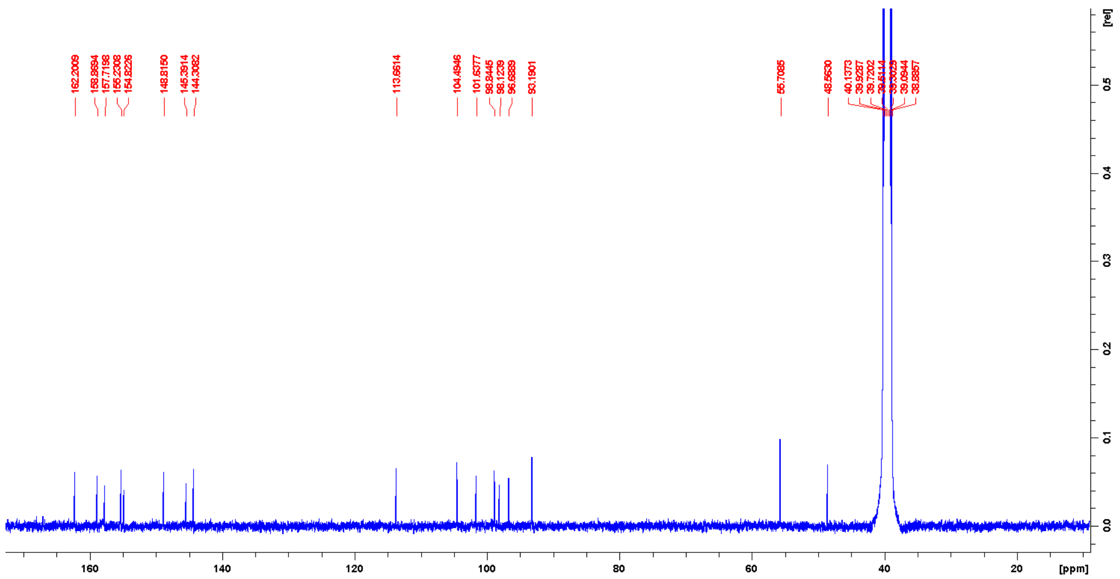

Figure S18 ^{13}C NMR (150 MHz, DMSO-d_6) spectrum of WED

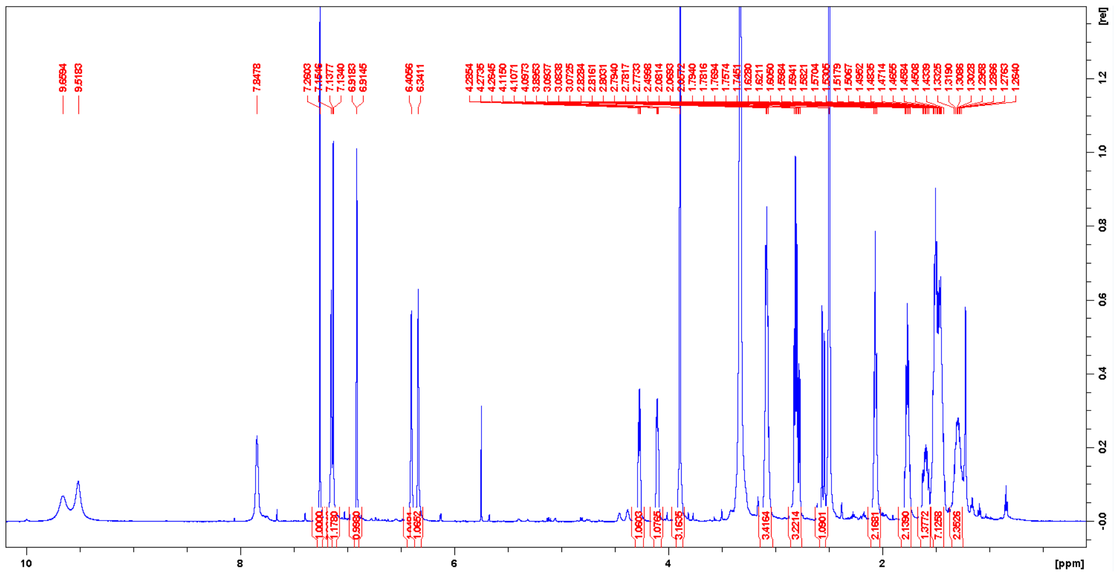

Figure S19 ^{1}H NMR (600 MHz, DMSO-d_6) spectrum of Bio-WED

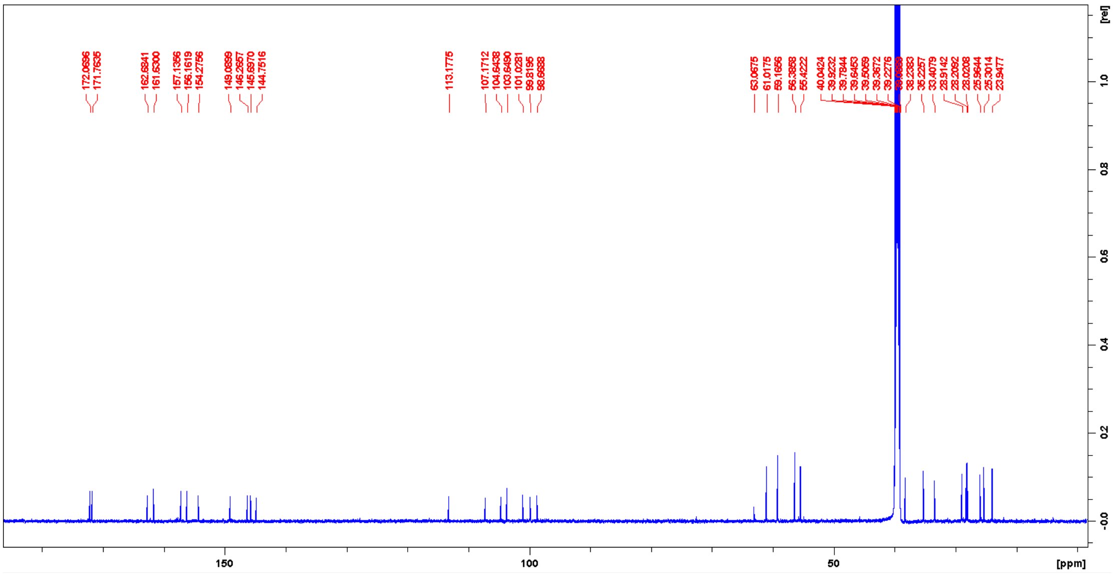

Figure S20 ^{13}C NMR (150 MHz, DMSO-d_6) spectrum of Bio-WED

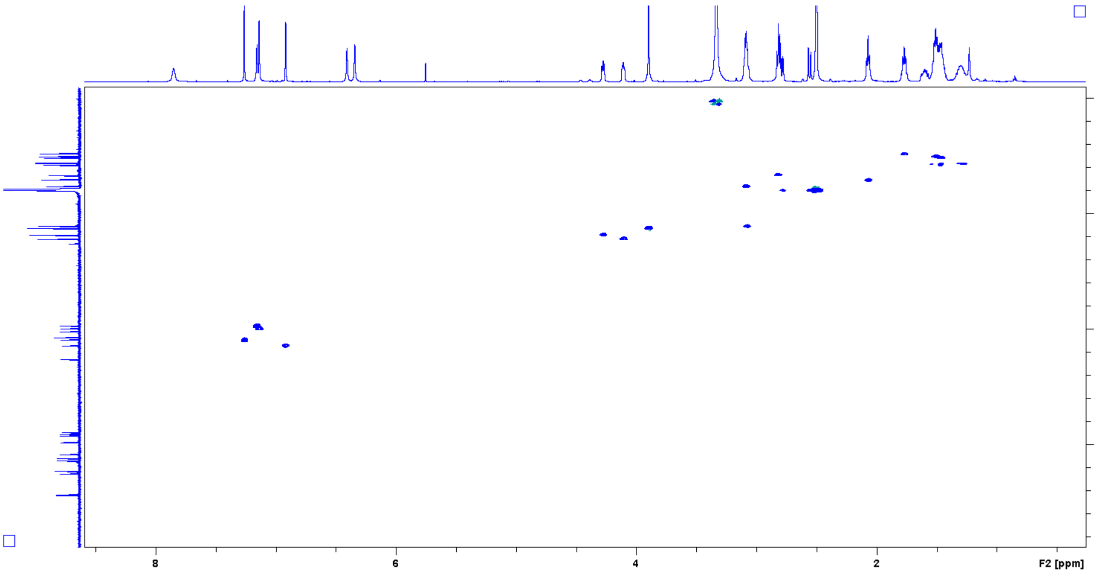

Figure S21 HSQC spectrum (600 MHz, DMSO-d_6) spectrum of Bio-WED

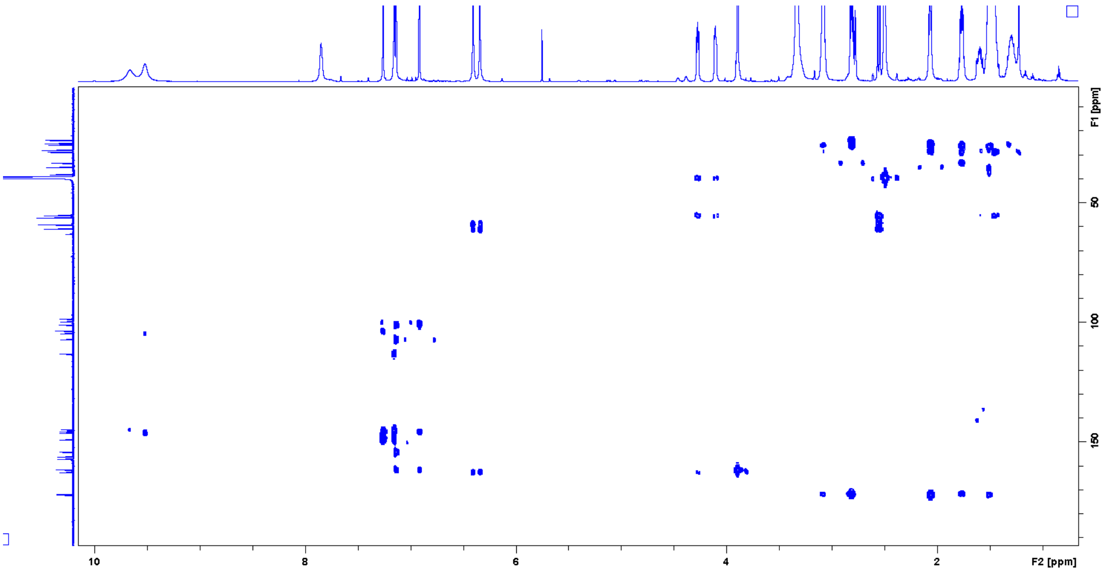

Figure S22 HMBC spectrum (600 MHz, DMSO-d_6) spectrum of Bio-WED
