# Supplementary material for: Macrophage Inactivation by Small Molecule Wedelolactone via Targeting sEH for the Treatment of LPS-Induced Acute Lung Injury
Source: ACS Cent Sci. 2023 Feb 21;9(3):440–56. doi: 10.1021/acscentsci.2c01424 (PMC10037491; doi:10.1021/acscentsci.2c01424)
Supplement: Supplementary file 2 — oc2c01424_si_002.pdf [file oc2c01424_si_002.pdf]

oc-2022-01424m.R1

Name: Peer Review Information for "Macrophage inactivation by small molecule wedelolactone via targeting sEH for the treatment of LPS-induced acute lung injury"

First Round of Reviewer Comments

Reviewer: 1

Comments to the Author

In this study, the authors reported that a small molecule wedelolactone (WED) targeted sEH leading to macrophage inactivation. Through the molecular interaction with amino acids Phe362 and Gln384, WED suppressed sEH activity to enhance levels of EETs thus attenuating inflammation and oxidative stress by regulatingGSK3beta-mediated NF-kB and Nrf2 pathways in vitro. In an LPS-stimulated ALI animal model, pharmacological sEH inhibition by WED or sEH KO alleviated pulmonary damage, such as the increase in the alveolar wall thickness and collapse. Additionally, WED or sEH genetic KO both suppressed macrophage activation, and attenuated inflammation and oxidative stress in vivo. These findings provided the broader prospects for ALI treatment by targeting sEH to alleviate inflammation and oxidative stress. many experiments were conducted well. This article is well written. I have some minor comments.

1) Methods: Catalog number of antibodies used in this study should be included as supplemental materials.

2) Figure legends: Please include the results (F-value with df, and P-values) of statistical analysis (one-way ANOVA, two-way ANOVA) in the legend or supplemental materials.

Reviewer: 2

Comments to the Author

This is an interesting study. The authors reported that WED attenuated LPS-induced ALI and macrophage inflammation by targeting sEH. Importantly, authors gave solid data that WED inhibited sEH. While I still have some comments as following:

1. Does WED inhibits sEH both at human and mice?

2. How inhibition of sEH contributes to the decrease in GSK3β? EETs?

3. Does sEH expression upregulate during ALI?

4. The effects of sEHI on macrophages inflammation and ALI have been reported, such as PMID: 27753791 and PMID: 32452554. The authors should discuss it in this manuscript.

Author's Response to Peer Review Comments:

Dear editor,

Thank you for your letter and the reviewers' comments concerning our manuscript (Manuscript ID: oc-2022-01424m). Those comments are all valuable and very helpful for revising and improving our paper. We have studied the comments carefully and have made corrections which we hope meet with approval. Revised descriptions are marked in yellow in the paper. The main corrections in the paper and the responses to the reviewer's comments are as follows.

We are looking forward to hearing from you at your earliest convenience.

Yours sincerely,

Cheng-Peng Sun,

College of Pharmacy, Dalian Medical University, No. 9, South Road of Lvshun, Dalian, 116044, China. Tel/Fax: +86-0411-86110419. E-mail: suncp146@163.com.

Reviewer(s)' Comments to Author:

Comments of reviewer 1:

Reviewer: 1

Recommendation: Publish in ACS Central Science after minor revisions noted.

Comments:

In this study, the authors reported that a small molecule wedelolactone (WED) targeted sEH leading to macrophage inactivation. Through the molecular interaction with amino acids Phe362 and Gln384, WED suppressed sEH activity to enhance levels of EETs thus attenuating inflammation and oxidative stress by regulatingGSK3beta-mediated NF-kB and Nrf2 pathways in vitro. In an LPS-stimulated ALI animal model, pharmacological sEH inhibition by WED or sEH KO alleviated pulmonary damage, such as the increase in the alveolar wall thickness and collapse. Additionally, WED or sEH genetic KO both suppressed macrophage activation, and attenuated inflammation and oxidative stress in vivo. These findings provided the broader prospects for ALI treatment by targeting sEH to alleviate inflammation and oxidative stress. many experiments were conducted well. This article is well written. I have some minor comments.

1) Methods: Catalog number of antibodies used in this study should be included as supplemental materials.

Response: Thank you very much for your comments. Catalog number of antibodies were added in the section of MATERIALS and METHODS.

The primary antibodies for sEH (10833-1-AP), NQO-1 (A1518), GSK3β (A2081), p-GSK3β (5558), TNF-α (17590-1-AP), IL-6 (21865-1-AP), iNOS (13120), MCP-1 (25542-1-AP), COX-2 (12282), p65 (8242), p-p65 (3033), Mfn1 (13798-1-AP), Mfn2 (12186-1-AP), HO-1 (A19062), Drp1 (12957-1-AP), GCLC (12601-1-AP), Opa1 (27733-1-AP), GCLM (14241-1-AP), Fis1 (10956-1-AP), Nrf2 (16396-1-AP), and Keap1 (10503-2-AP) were purchased from Cell Signaling Technology (CST, Danvers, MA, USA), Abclonal (Wuhan, China), Proteintech (Wuhan, China), Abcam (Massachusetts, USA), and Affinity (Cincinnati, USA).

2) Figure legends: Please include the results (F-value with df, and P-values) of statistical analysis (one-way ANOVA, two-way ANOVA) in the legend or supplemental materials.

Response: Thank you very much for your comments. F-value with df, and P-values were added in the legend or supplemental materials.

Figure 1 WED alleviated inflammatory responses *in vitro* through the NF-κB pathway. (A) WED suppressed the release of LPS-induced TNF-α (*p* < 0.0001, DF = 4, 10, F-value = 143.3) and IL-6 (*p* < 0.0001, DF = 4, 10, F-value = 238.1) in RAW264.7 cells (mean ± SEM, n = 3, one-way ANOVA). (B) WED downregulated mRNA levels of *TNF-α* (*p* < 0.0001, DF = 4, 10, F-value = 71.86), *IL-6* (*p* < 0.0001, DF = 4, 10, F-value = 147.1), *COX-2* (*p* < 0.0001, DF = 4, 10, F-value = 79.7), *iNOS* (*p* < 0.0001, DF = 4, 10, F-value = 33.1), *MCP-1* (*p* < 0.0001,

DF = 4, 10, F-value = 21.1), and *ICAM-1* (*p* = 0.0019, DF = 4, 10, F-value = 9.6) in LPS-induced RAW264.7 cells (mean ± SEM, n = 3, one-way ANOVA). (C) WED inactivated the NF-κB pathway (*p* < 0.0001, DF = 4, 10, F-value = 28.2) to downregulate expression levels of its target proteins TNF-α (*p* < 0.0001, DF = 4, 10, F-value = 29.6), IL-6 (*p* < 0.0001, DF = 4, 10, F-value =31.1), COX-2 (*p* < 0.0001, DF = 4, 10, F-value = 105.5), iNOS (*p* < 0.0001, DF = 4, 10, F-value = 41.8), and MCP-1 (*p* < 0.0001, DF = 4, 10, F-value = 23.7) in LPS-induced RAW264.7 cells (mean ± SEM, n = 3, one-way ANOVA). (D and E) WED suppressed the translocation of p65 to the nucleus analyzed by the confocal microscopy (D) and Western blot (E, mean ± SEM, n = 3, one-way ANOVA; For p65 in nucleus, *p* < 0.0001, DF = 4, 10, F-value =30.3; For p65 in cytoplasm, *p* = 0.0015, DF = 4, 10, F-value = 10.2).

Figure 2 WED alleviated the oxidative reduction in vitro through the Nrf2 pathway. (A) WED enhanced levels of GSH (*p* = 0.0018, DF = 4, 10, F-value = 9.8) and SOD (*p* = 0.0002, DF = 4, 10, F-value = 17.1) in LPS-induced RAW264.7 cells (mean ± SEM, n = 3, one-way ANOVA). (B) The flow cytometry demonstrated that WED reduced the LPS-induced ROS positive cells. (C) Quantitative data of ROS positive cells in LPS-stimulated RAW264.7 cells treated with WED (mean ± SEM, n = 3, one-way ANOVA, *p* < 0.0001, DF = 4, 10, F-value = 24.0). (D) WED reversed the increase of mtDNA content in LPS-induced RAW264.7 cells (mean ± SEM, n = 3, one-way ANOVA, *p* < 0.0001,

DF = 4, 10, F-value = 27.6). (E) The fluorometric analysis indicated that WED suppressed the ROS production. (F) WED reversed the effect of LPS-mediated mitochondrial membrane potential. (G) WED regulated expressions of proteins Mfn1, Mfn2, Op1, Drp1, and Fis1 involved in the mitochondrial fusion and fission. (H) WED activated the Nrf2 pathway to regulate expression levels of HO-1, NQO-1, GCLC, GCLM, Nrf2, and Keap1. (I and K) WED suppressed the translocation of Nrf2 to the nuclear analyzed by Western blot (I, mean ± SEM, n = 3, one-way ANOVA; For Nrf2 in nucleus, $p < 0.0001$, DF = 4, 10, F-value = 38.9; For Nrf2 in cytoplasm, $p = 0.0001$, DF = 4, 10, F-value = 18.67) and the confocal microscopy (K). (J) WED regulated mRNA levels of genes *Keap1* ($p < 0.0001$, DF = 4, 10, F-value = 202.5), *HO-1* ($p = 0.0002$, DF = 4, 10, F-value = 16.5), *NQO-1* ($p < 0.0001$, DF = 4, 10, F-value = 84.5), and *Nrf2* ($p < 0.0001$, DF = 4, 10, F-value = 30.0) involved in the Nrf2 pathway (mean ± SEM, n = 3, one-way ANOVA). (L) The luciferase assay demonstrated the activation of WED against the Nrf2 receptor (mean ± SEM, n = 5, one-way ANOVA, $p < 0.0001$, DF = 3, 16, F-value = 65.6).

Figure 3 WED attenuated the course of ALI in LPS-induced ALI mice. (A) Representative H&E staining plots. (B) Representative CD68 staining plots. (C) Representative Gr-1 staining plots. (D) WED attenuated the infiltration of proteins ($p < 0.0001$, DF = 5, 42, F-value = 11.3), WBCs ($p < 0.0001$, DF = 5, 42, F-value = 36.2), PMNs ($p < 0.0001$, DF = 5, 42, F-value = 57.8), and MNs ($p < 0.0001$, DF = 5, 42, F-value = 32.1) to the pulmonary alveoli, and reduced the production of IL-6 ($p < 0.0001$, DF = 5, 42, F-value = 31.8) and TNF-α ($p <$

0.0001, DF = 5, 42, F-value = 41.2) and the activity of MPO (*p* < 0.0001, DF = 5, 42, F-value = 9.8) and LDH (*p* < 0.0001, DF = 5, 42, F-value = 27.7) in LPS-induced ALI mice (mean ± SEM, n = 8, one-way ANOVA). (E) WED reduced the production of IL-6 (*p* < 0.0001, DF = 5, 42, F-value = 23.8) and TNF-α (*p* < 0.0001, DF = 5, 42, F-value = 14.2) and the activity of MPO (*p* < 0.0001, DF = 5, 42, F-value = 27.6) and LDH (*p* < 0.0001, DF = 5, 42, F-value = 7.9) in the lung of LPS-induced ALI mice (mean ± SEM, n = 8, one-way ANOVA).

Figure 4 WED attenuated the inflammation and oxidative stress *in vivo*. (A) Representative TNF-α staining plots. (B) Effects of WED against inflammatory genes *TNF-α* (*p* < 0.0001, DF = 5, 24, F-value = 30.5), *IL-6* (*p* < 0.0001, DF = 5, 24, F-value = 56.7), *COX-2* (*p* < 0.0001, DF = 5, 24, F-value = 12.8), *iNOS* (*p* < 0.0001, DF = 5, 24, F-value = 21.0), *ICAM-1* (*p* < 0.0001, DF = 5, 24, F-value = 21.4), and *MCP-1* (*p* < 0.0001, DF = 5, 24, F-value = 25.5) in LPS-induced ALI mice (mean ± SEM, n = 5, one-way ANOVA). (C) WED suppressed the NF-κB pathway to downregulate expression levels of its target proteins IL-6, COX-2, and MCP-1. (D) Representative scanning electron microscope plots. (E) Effects of WED toward the MDA level (*p* < 0.0001, DF = 5, 42, F-value = 13.4) and the SOD activity (*p* < 0.0001, DF = 5, 42, F-value = 15.4) in LPS-induced ALI mice (mean ± SEM, n = 8, one-way ANOVA). (F) WED reversed the increase of the mtDNA content in LPS-induced ALI mice (mean ± SEM, n = 8, one-way ANOVA, *p* < 0.0001, DF = 5, 24, F-value = 20.3). (G) WED regulated expressions of proteins Mfn1, Mfn2, Op1, Drp1, and Fis1 involved in the mitochondrial fusion and fission in LPS-induced ALI mice. (H) Representative Nrf2 staining plots. (I) WED activated the Nrf2 pathway to regulate expression levels of HO-1, NQO-1, GCLC, GCLM, Nrf2, and Keap1 in LPS-induced ALI mice.

Figure 5 sEH served as the direct target of WED in the anti-inflammation and antioxidation. (A) Identification of the cellular target of WED using the

pull-down technology based on the WED-coupled Sepharose 6B beads and the LC–MS/MS analysis. (B) The LC-MS/MS plot of sEH. (C) The binding protein was detected by Western blot. (D) The IP-MS analysis indicated the interaction of WED with sEH. (E) CETSA and DARTS results. (F) Quantitative data of CETSA and DARTS (mean ± SEM, n = 3, one-way ANOVA, *p* < 0.0001, DF = 5, 12, F-value = 100.2). (G) The MST result of WED with sEH (mean ± SEM, n = 3). (H) The scheme of Bio-WED. (I) The co-location of WED with sEH detected by the fluorescence microscope. (J) The CYP/sEH-mediated AA metabolism pathway. (K) The binding capability of WED with sEH detected by the fluorescence-based binding assay (mean ± SEM, n = 3). (L) The inhibitory effect of WED against the sEH activity detected by the system of human recombinant sEH-mediated hydrolysis of the substrate PHOME (mean ± SEM, n = 3). (M) WED suppressed the sEH activity (14,15-EET/14,15-DHET, *p* < 0.0001, DF = 4, 10, F-value = 55.89) analyzed by levels of 14,15-EET (*p* = 0.0003, DF = 4, 10, F-value = 15.1) and its corresponding diol (*p* = 0.0034, DF = 4, 10, F-value = 8.2) in LPS-mediated RAW264.7 cells (mean ± SEM, n = 3, one-way ANOVA).

Figure 6 sEH knockdown and rescue abolished anti-inflammatory and antioxidant effects of WED in vitro. (A) sEH knockdown abolished effects of WED toward inflammatory and antioxidant genes *TNF-α* (*p* = 0.0028, DF = 1, 8, F-value = 18.0), *IL-6* (*p* < 0.0001, DF = 1, 8, F-value = 355.1), *COX-2* (*p* = 0.0058, DF = 1, 8, F-value = 13.9), *iNOS* (*p* < 0.0001, DF = 1, 8, F-value = 133.8), *NQO-1* (*p* = 0.0037, DF = 1, 8, F-value = 16.4), *Nrf2* (*p* = 0.0194, DF = 1, 8, F-value =8.5), and *Keap1* (*p* = 0.0006, DF = 1, 8, F-value = 30.0) in LPS-induced RAW264.7 cells (mean ± SEM, n = 3, two-way ANOVA). (B) sEH knockdown abolished effects of WED toward the NF-κB and Nrf2 pathways measured by Western blot. (C) sEH rescue weakened effects of WED toward inflammatory and antioxidant genes *TNF-α* (*p* = 0.0006, DF = 1, 8, F-value = 29.7), *IL-6* (*p* = 0.0921, DF = 1, 8, F-value =3.7), *COX-2* (*p* = 0.0489, DF = 1, 8, F-value = 5.4), *iNOS* (*p* = 0.4830, DF = 1, 8, F-value = 0.5), *NQO-1* (*p* = 0.6083, DF = 1, 8, F-value = 0.3), *HO-1* (*p* = 0.8389,

DF = 1, 8, F-value = 0.04), and *Nrf2* (*p* = 0.8812, DF = 1, 8, F-value = 0.02) in LPS-induced RAW264.7 cells (mean ± SEM, n = 3, two-way ANOVA). (D) sEH rescue weakened effects of WED toward the NF-κB and Nrf2 pathways measured by Western blot.

Figure 7 Phe362 and Gln384 played roles in the binding of WED with sEH. (A) The RMSD plot analyzed by molecular dynamics. (B) Effect of WED against the volume of the pocket. (C) The trajectories of Phe362, Ile363, Ser374, and Asn378 with WED. (D) The trajectories of Tyr363, Gln384, Gln502, and Met503 with WED. (E) The interactions of WED with sEH through hydrogen bonds of Phe362 and Gln384. (F) Phe362Ala and Gln384Ala mutations abolished the binding of WED with sEH. (G) Phe362Ala and Gln384Ala mutations abolished anti-inflammatory (*IL-6*, *p* = 0.0014, DF = 2, 12, F-value = 12.0; *TNF-α*, *p* < 0.0001, DF = 2, 12, F-value = 37.4; *COX-2*, *p* = 0.0005, DF = 2, 12, F-value = 15.1) and anti-oxidative effects (*HO-1*, *p* = 0.0001, DF = 2, 12, F-value = 20.6; *Nrf2*, *p* < 0.0001, DF = 2, 12, F-value = 30.5) of WED in LPS-stimulated RAW264.7 cells (mean ± SEM, n = 3, two-way ANOVA).

Figure 8 GSK3β is the downstream key pathway of sEH in the anti-inflammatory and antioxidant effects of WED. (A) sEH knockdown suppressed the GSK3β activity. (B) sEH rescue activated the GSK3β activity. (C) Inhibition of sEH by WED suppressed the GSK3β activity. (D) Inhibition of GSK3β by LiCl abolished effects of WED toward inflammatory and antioxidant genes *TNF-α* (*p* = 0.0316, DF = 1, 8, F-value = 6.8), *IL-6* (*p* = 0.0006, DF = 1, 8, F-value = 29.8), *COX-2* (*p* < 0.0001, DF = 1, 8, F-value =52.5), *iNOS* (*p* = 0.0001, DF = 1, 8, F-value = 50.2), *HO-1* (*p* = 0.0097, DF = 1, 8, F-value = 11.4), *NQO-1* (*p* = 0.0005, DF = 1, 8, F-value = 32.2), and *Nrf2* (*p* < 0.0001, DF = 1, 8, F-value = 55.7) *in vitro* (mean ± SEM, n = 3, two-way ANOVA). (E) Inhibition of GSK3β by LiCl abolished effects of WED toward the NF-κB (*p* = 0.0154, DF = 1, 8, F-value = 9.4) and Nrf2 (*p* < 0.0001, DF = 1, 8, F-value = 69.8) pathways measured by

Western blot (mean ± SEM, n = 3, two-way ANOVA).

Figure 9 WED suppressed the sEH activity to allow the inhibition of GSK3β *in vivo*. (A) Heat map of sEH substrates (e.g. 8,9-EET, 11,12-EET, and 14,15-EET) and their corresponding diols (e.g. 8,9-DHET, 11,12-DHET, and 14,15-DHET). (B) WED reduced the ratio of DHETs and EETs (8,9-DHET/8,9-EET, $p < 0.0001$, DF = 5, 30, F-value = 20.5; 11,12-DHET/11,12-EET, $p < 0.0001$, DF = 5, 30, F-value = 37.5; 14,15-DHET/14,15-EET, $p < 0.0001$, DF = 5, 30, F-value = 22.9) *in vivo* to suppress the sEH activity (mean ± SEM, n = 6, one-way ANOVA). (C) Inhibition of sEH by WED led to the inactivation of the GSK3β activity ($p < 0.0001$, DF = 5, 12, F-value = 25.3) in LPS-induced ALI mice (mean ± SEM, n = 3, one-way ANOVA).

Figure 10 sEH genetic KO abolished the pulmonary protective effect of WED. (A) Genotyping and confirmation of sEH knockout mice. (B and C) Representative plots of H&E (B), CD68, and Gr-1 (C) staining in LPS-induced ALI *Ephx2*$^{+/+}$ and *Ephx2*$^{-/-}$ mice treated with WED. (D) Measurement of pulmonary MPO ($p < 0.0001$, DF = 1, 20, F-value = 32.7), TNF-α ($p = 0.0323$, DF = 1, 20, F-value = 5.3), and IL-6 ($p = 0.0002$, DF = 1, 20, F-value = 20.5) from LPS-induced ALI *Ephx2*$^{+/+}$ and *Ephx2*$^{-/-}$ mice treated with WED (mean ± SEM, n = 6, two-way ANOVA). (E) *Ephx2* KO abolished the effect of WED on the inhibition of GSK3β ($p = 0.0019$, DF = 1, 8, F-value = 20.4) in LPS-induced ALI (mean ± SEM, n = 3, two-way ANOVA)

Figure 11 sEH genetic KO abolished anti-inflammatory and antioxidant effects of WED. (A) Representative TNF-α staining plots in LPS-induced ALI *Ephx2*$^{+/+}$ and *Ephx2*$^{-/-}$mice treated with WED. (B) *Ephx2* KO abolished the effect of WED on expressions of COX-2 and p-p65/p65 in LPS-induced ALI. (C) *Ephx2* KO abolished the effect of WED on mRNA levels of *COX-2* ($p = 0.0267$, DF = 1, 16, F-value = 6.0), *iNOS* ($p = 0.0033$, DF = 1, 16, F-value = 11.9), *TNF-α* ($p = 0.0058$,

DF = 1, 16, F-value = 10.1), *IL-6* (*p* = 0.0003, DF = 1, 16, F-value = 21.6), and *ICAM-1* (*p* = 0.0842, DF = 1, 16, F-value = 3.4) in LPS-induced ALI (mean ± SEM, n = 5, two-way ANOVA). (D) Representative HO-1 and Nrf2 staining plots in LPS-induced ALI *Ephx2*$^{+/+}$ and *Ephx2*$^{-/-}$ mice treated with WED. (E) Measurement of pulmonary MDA (*p* = 0.0012, DF = 1, 20, F-value = 14.3), GSH (*p* = 0.0008, DF = 1, 20, F-value = 15.5), and SOD (*p* = 0.0012, DF = 1, 20, F-value = 14.4) from LPS-induced ALI *Ephx2*$^{+/+}$ and *Ephx2*$^{-/-}$ mice treated with WED (mean ± SEM, n = 6, two-way ANOVA). (F) *Ephx2* KO abolished the effect of WED on expressions of Mfn1, Drp1, HO-1, and Nrf2 in LPS-induced ALI. (G) *Ephx2* KO abolished the effect of WED on mRNA levels of *NQO-1* (*p* = 0.0047, DF = 1, 16, F-value = 10.8) and *Nrf2* (*p* = 0.0041, DF = 1, 16, F-value = 11.2) in LPS-induced ALI (mean ± SEM, n = 5, two-way ANOVA).

Comments of reviewer 2:

Recommendation: Reconsider after major revisions noted.

Comments:

This is an interesting study. The authors reported that WED attenuated LPS-induced ALI and macrophage inflammation by targeting sEH. Importantly, authors gave solid data that WED inhibited sEH. While I still have some comments as following:

1. Does WED inhibits sEH both at human and mice?

Response: Thank you very much for your comments. We have detected the ability of WED toward human and mouse sEH, and found that WED could inhibit human and mouse sEH activities with IC_{50} values of 3.06 and 1.27 μM. These data were added in Figure 5L and S9.

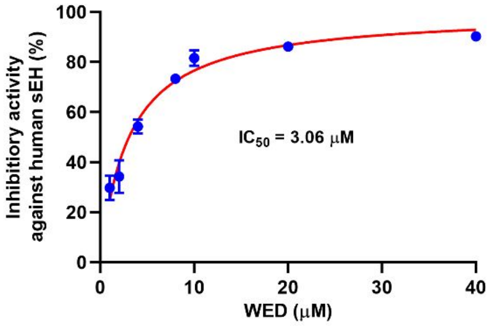

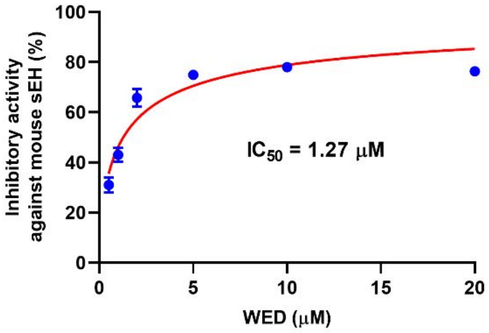

2. How inhibition of sEH contributes to the decrease in GSK3β? EETs?

Response: Thank you very much for your comments. Inhibition of sEH could regulate the GSK3β activity through EET according to our results.

We performed the experiment focused on effects of inhibition of sEH toward the GSK3β activity with or without 14,15-EET. We found that 14,15-EET decreased the GSK3β activity, and abolished the effect of inhibition of sEH by WED toward GSK3β, demonstrating that sEH inhibition regulated the GSK3β pathway through EET, which was supported by previous reports (Food and Chemical Toxicology 156 (2021) 112516; PNAS, 2022, 119, e2118818119). These data were added in Supporting information (Figure S13).

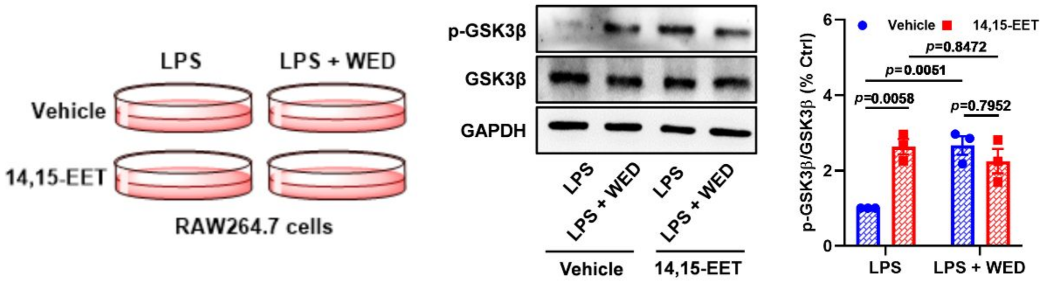

Figure S13 14,15-EET abolished the effect of WED toward the GSK3β activity *in vitro* (*p* = 0.0020, DF = 1, 8, F-value = 20.3).

3. Does sEH expression upregulate during ALI?

Response: Thank you very much for your comments. We have detected the level of sEH in ALI, and found that sEH were upregulated in ALI. These data were added in Supporting information (Figure S14).

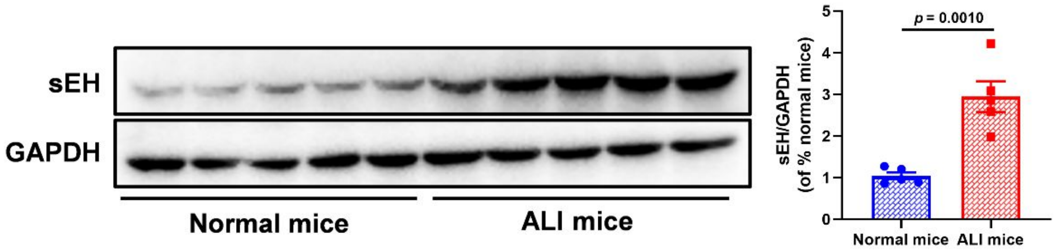

Figure S14 Overexpression of sEH in ALI mice (mean ± SEM, n = 5).

4. The effects of sEHI on macrophages inflammation and ALI have been reported, such as PMID: 27753791 and PMID: 32452554. The authors should discuss it in this manuscript.

Response: Thank you very much for your comments. We have revised the discussion section and these two key references (PMID: 27753791 and PMID: 32452554) have been added in this section according to your comments. The references have been cited and added in the manuscript.

Although the ability to define drug target protein recognition technology is rapidly developing, the discovery of drug targets still faces many challenges [41]. Recent researches have reported the application of the affinity chromatography, biotin-avidin, and ligand-induced stability shift-dependent proteomics technologies for discovering the direct target of natural products, such as kurarinone, alisol B, eupalinolide B, and handelin [24, 42, 43]. In this study, we uncovered that sEH was the direct cellular target of WED based on technologies employing affinity chromatography. The sEH is a bifunctional enzyme with the

C-terminal hydrolase and N-terminal phosphatase activities. The sEH is responsible for the hydrolysis of the bioactive metabolites EETs transformed by CYP2J, CYP2C and other CYP enzyme from AA to their corresponding epoxides [14, 23]. Increasing evidence has revealed the role of sEH in inflammation and oxidative stress-mediated lung diseases [46-48]. For example, chemical inhibition of sEH by 1-trifluoromethoxyphenyl-3-(1-propionylpiperidin-4-yl) urea (TPPU) suppressed LPS-mediated macrophage activation in vitro to regulate the pro-inflammatory cytokine level [49-51], increased the survival rate of LPS-induced ALI, and attenuated the neutrophil infiltration and alveolar capillary leakage in ALI animal model [49, 50]. Furthermore, Ephx2 genetic KO attenuated the pulmonary inflammation and edema in hyperoxia- or cigarette smoke-mediated lung injury mice [46-48], suggesting the potential of sEH in macrophage activation-induced ALI. As expected, sEH knockdown alleviated the inflammation and oxidative stress, and the sEH rescue magnified the abovementioned changes in vitro. Similarly, sEH KO remarkably attenuated the course of ALI in an LPS-induced mouse model. Furthermore, the protective effect of WED was abolished by sEH knockdown, KO, and rescue.

(49) Luo, X. Q.; Duan, J. X.; Yang, H. H.; Zhang, C. Y.; Sun, C. C.; Guan, X. X.; Xiong, J. B.; Zu, C.; Tao, J. H.; Zhou, Y.; Guan, C. X. Epoxyeicosatrienoic acids inhibit the activation of NLRP3 inflammasome in murine macrophages. J Cell Physiol 2020, 235 (12), 9910-9921.

(50) Zhou, Y.; Liu, T.; Duan, J. X.; Li, P.; Sun, G. Y.; Liu, Y. P.; Zhang, J.; Dong, L.; Lee, K. S. S.; Hammock, B. D.; Jiang, J. X.; Guan, C. X. Soluble Epoxide Hydrolase Inhibitor Attenuates Lipopolysaccharide-Induced Acute Lung Injury and Improves Survival in Mice. Shock 2017, 47 (5), 638-645.

(51) Dong, L.; Zhou, Y.; Zhu, Z. Q.; Liu, T.; Duan, J. X.; Zhang, J.; Li, P.; Hammcok, B. D.; Guan, C. X. Soluble Epoxide Hydrolase Inhibitor Suppresses the Expression of Triggering Receptor Expressed on Myeloid Cells-1 by Inhibiting NF-kB Activation in Murine Macrophage. Inflammation 2017, 40 (1), 13-20.

oc-2022-01424m.R2

Name: Peer Review Information for "Macrophage inactivation by small molecule wedelolactone via targeting sEH for the treatment of LPS-induced acute lung injury"

Second Round of Reviewer Comments

Reviewer: 1

Comments to the Author

My comments have been addressed.

Reviewer: 2

Comments to the Author

Thank you very much for the explanation. I have no question.

Author's Response to Peer Review Comments:

Dear editor,

Thanks for your letter. We have revised format, and added Supporting information section and synopsis in the manuscript as follows:

Supporting Information

The Supporting Information is available free of charge at

Supplementary Figures S1-22.

Targeting sEH with WED to enhance levels of EETs suppressed the macrophage activation via regulating GSK3beta-mediated NF-kB and Nrf2 pathways, resulting in the attenuation of LPS-induced ALI.

Best wishes,

Cheng-Peng Sun

Dear editor,

Thank you for your letter and the reviewers' comments concerning our manuscript (Manuscript ID: oc-2022-01424m). Those comments are all valuable and very helpful for revising and improving our paper. We have studied the comments carefully and have made corrections which we hope meet with approval.

We have revised format, and added Supporting information section in the manuscript as follows:

Supporting Information

The Supporting Information is available free of charge at

Supplementary Figures S1-22.

The synopsis "Targeting sEH with WED to enhance levels of EETs suppressed the macrophage activation via regulating GSK3beta-mediated NF-kB and Nrf2 pathways, resulting in the attenuation of LPS-induced ALI." has been added in the manuscript file after the TOC graphic.

We are looking forward to hearing from you at your earliest convenience.

Yours sincerely,

Cheng-Peng Sun,

College of Pharmacy, Dalian Medical University, No. 9, South Road of Lvshun, Dalian, 116044, China. Tel/Fax: +86-0411-86110419. E-mail: suncp146@163.com.
